# On brain atlas choice and automatic segmentation methods: a comparison of MAPER & FreeSurfer using three atlas databases

**DOI:** 10.1038/s41598-020-57951-6

**Published:** 2020-02-18

**Authors:** Siti Nurbaya Yaakub, Rolf A. Heckemann, Simon S. Keller, Colm J. McGinnity, Bernd Weber, Alexander Hammers

**Affiliations:** 1 King’s College London & Guy’s and St Thomas’ PET Centre, School of Biomedical Engineering & Imaging Sciences, King’s College London, London, United Kingdom; 2 MedTech West at Sahlgrenska University Hospital Gothenburg, Gothenburg, Sweden; 3 Department of Radiation Physics, Institute of Clinical Sciences, Gothenburg University, Gothenburg, Sweden; 4 Division of Brain Sciences, Imperial College London, London, United Kingdom; 5 Department of Molecular and Clinical Pharmacology, Institute of Translational Medicine, University of Liverpool, Liverpool, United Kingdom; 6 Department of Neuroradiology, The Walton Centre NHS Foundation Trust, Liverpool, United Kingdom; 7 Center for Economics and Neuroscience, University of Bonn, Bonn, Germany; 8 Institute of Experimental Epileptology and Cognition Research, University Hospital Bonn, Bonn, Germany

**Keywords:** Alzheimer's disease, Brain, Epilepsy

## Abstract

Several automatic image segmentation methods and few atlas databases exist for analysing structural T1-weighted magnetic resonance brain images. The impact of choosing a combination has not hitherto been described but may bias comparisons across studies. We evaluated two segmentation methods (MAPER and FreeSurfer), using three publicly available atlas databases (Hammers_mith, Desikan-Killiany-Tourville, and MICCAI 2012 Grand Challenge). For each combination of atlas and method, we conducted a leave-one-out cross-comparison to estimate the segmentation accuracy of FreeSurfer and MAPER. We also used each possible combination to segment two datasets of patients with known structural abnormalities (Alzheimer’s disease (AD) and mesial temporal lobe epilepsy with hippocampal sclerosis (HS)) and their matched healthy controls. MAPER was better than FreeSurfer at modelling manual segmentations in the healthy control leave-one-out analyses in two of the three atlas databases, and the Hammers_mith atlas database transferred to new datasets best regardless of segmentation method. Both segmentation methods reliably identified known abnormalities in each patient group. Better separation was seen for FreeSurfer in the AD and left-HS datasets, and for MAPER in the right-HS dataset. We provide detailed quantitative comparisons for multiple anatomical regions, thus enabling researchers to make evidence-based decisions on their choice of atlas and segmentation method.

## Introduction

Accurate segmentation of T1-weighted magnetic resonance (MR) brain images into anatomical regions is a regular prerequisite of quantitative analysis. Brain morphometric features, such as regional volume, have been used to describe and distinguish development stages and disease states. As an alternative to labour-intensive expert manual labelling, automatic methods such as FreeSurfer and MAPER (multi-atlas propagation with enhanced registration) are widely used to label novel target images.

Both methods are based on the principal idea of transferring knowledge from an atlas to a target image. An atlas in this context is the combination of an image and a trusted reference segmentation. Reference segmentations are typically generated by experts following a pre-established delineation protocol. The accuracy of the target segmentation depends crucially on the accuracy of the atlas segmentations.

Manual segmentation does not scale well, and only automatic methods have enabled the analysis of modern large datasets such as ADNI^1^. FreeSurfer is a widely used software suite that enables fully-automated surface-based cortical segmentation as well as subcortical volume-based segmentation^2–6^. MAPER is a software for automatic volumetric segmentation of brain MR images via multiple registrations of reference atlases, taking overall brain morphology (e.g. atrophy, wide ventricles) into account during the registrations themselves^7,8^.

MAPER and FreeSurfer have been independently validated against manual labels^4,5,7,8^, and have been compared against each other and other segmentation methods for specific, often sub-cortical brain regions in numerous studies^9–17^. However, it is unknown how different segmentation methods compare to each other when tasked with automatically segmenting both cortical and sub-cortical regions across the whole brain, which facilitates machine-learning applications^18–21^.

Most automatic methods are coupled to specific atlases. We use the term “native atlas” to refer to an atlas that is tied to a segmentation method through historical co-development and/or bundled distribution. The atlases that are packaged with FreeSurfer are optimized for surface parcellation, but the manual segmentations are not publicly available. MAPER is typically used with the volume-based Hammers_mith (HM) atlases, which are available online (http://brain-development.org) and were published with detailed delineation protocols^22–26^ and have been extended to infants^23^ and newborns^27,28^. Both FreeSurfer and MAPER enable users to apply another atlas database of their choosing^29^. It is, however, unknown how either of the methods perform when users apply non-native atlases.

Atlas choice is an important, but often overlooked aspect of neuroimaging analyses. The variety of available brain parcellation and segmentation protocols reflects the variety of purposes and motivations for constructing atlases^30^: cytoarchitectonic^31–33^, landmark-based^22,34^, varying degrees of subdivision^25,35^, functional and connectivity-based parcellations^36–38^ and multi-modal parcellations^39^. Comparing atlases is non-trivial also because of the diversity of subjects and subject groups used. Some atlases are based on single-subject images, such as the Automated Anatomical Labelling (AAL) atlas^40^, and do not capture inter-individual variability. The choice of atlas has implications for interpretation, for comparisons across studies and populations, and for use in meta-analyses.

In our work on quantitative characterization of neuroanatomical disease correlates, we generally use MAPER with the HM atlas^41–44^, since as creators and co-creators we are thoroughly familiar with the characteristics of this combination. To assess the cost/benefit that our bias entails, we sought to compare our preferred setup quantitatively with the most obvious (i.e. widely-used) alternative, FreeSurfer. To disentangle the effects of atlas quality from those of algorithm suitability, we decided to use each algorithm with each other’s atlas database (or a close approximation), i.e. Desikan-Killiany-Tourville (DKT) with MAPER and HM with FreeSurfer. While planning this experiment, we pondered the potential benefit of a full study, seeing that more general guidance on choosing a method-database combination would likely be useful to other scientists and practitioners. To provide such guidance, we additionally employed a “neutral” (independently developed) third atlas database, from the MICCAI 2012 Grand Challenge, for benchmarking purposes.

Further extending the scope, in addition to within-database leave-one-out cross-comparisons, we designed a comparison of each method-database combination’s ability to detect volumetric differences between disease and control groups contained in two independently acquired clinical study cohorts, one on Alzheimer’s disease and one on hippocampal sclerosis in temporal lobe epilepsy.

## Methods

### Atlas databases

We applied three publicly available atlas databases of healthy adult participants consisting of anonymized T1-weighted 3D MR images with corresponding manual or semi-automated segmentation labels.

The first atlas database was the Hammers_mith brain atlas^22,23,25,26^ (www.brain-development.org), which was one of the atlas sets used in the development of the MAPER software. This atlas set consists of 95 manually delineated regions drawn on T1-weighted images from 30 healthy young adult subjects. Regions in this atlas were manually drawn to include both grey and white matter. All manually drawn regions were checked by a neurologist. For compatibility with other atlases, cortical region labels and output segmentations were multiplied with a grey-matter mask. Brain extraction was performed by multiplying the MR image with a mask combined from the manual segmentations with FSL BET (https://fsl.fmrib.ox.ac.uk/fsl/fslwiki/BET) output in a manner that ensures matching surfaces of the manual segmentation and the extraction mask at the cortical surface. The detail of this procedure is described in Supplementary Methods. The grey-matter mask was obtained using FAST from the FSL suite^45^ (https://fsl.fmrib.ox.ac.uk/fsl/fslwiki/FAST). A three-tissue class (grey matter, white matter and cerebrospinal fluid) image was created by assigning each voxel to the tissue class having the maximum probability at that location. The grey-matter component of the three-tissue class image was used to mask all cortical regions. We chose FSL FAST for creating the grey-matter mask as this was the standard used in the MAPER segmentation software, which was co-developed with the Hammers_mith atlases. This atlas set will be referred to as the *HM atlas database*.

The second atlas database originated as the subset of the Mindboggle-101 database which underlies the Desikan-Killiany-Tourville (DKT) classifier atlas in the FreeSurfer package (http://www.mindboggle.info/data.html)^46^. The DKT classifier atlas database consists of 40 T1-weighted images from healthy adult subjects with 62 cortical surface labels (31 regions per hemisphere). The segmentations were generated from an initial automatic segmentation with FreeSurfer’s Desikan-Killiany atlas^34^, then manually edited according to the DKT protocol^46^ by a single investigator and subsequently checked by a senior scientist. Since segmentations were done on the cortical surface, the volumetric projections for each region included only the grey matter. This atlas set will be referred to as the *DKT40 atlas database*.

The third atlas database was independent of either of the two segmentation methods under consideration in this work. It was created for the MICCAI 2012 Grand Challenge and Workshop on Multi-Atlas Labelling (https://my.vanderbilt.edu/masi/workshops). It consists of T1-weighted MR images from 30 subjects from the OASIS database^47^ with 138 manually annotated cortical and sub-cortical structures provided by Neuromorphometrics, Inc. (http://neuromorphometrics.com). The first timepoint was used for subjects that were scanned twice. Segmentations were performed by neuroanatomical technicians according to the Neuromorphometrics’ General Segmentation Protocol (http://www.neuromorphometrics.org:8080/seg) and the BrainCOLOR Cortical Parcellation Protocol (https://www.binarybottle.com/braincolor/index.html) and subsequently checked by another technician or by a consulting anatomist. Two regions were excluded due to their small size (*cerebral exterior* and *vessel)*. Labelled regions relating to the cortex only included grey matter, since white matter was explicitly labelled in this atlas database, partly by a histogram method and partly by manual labelling (http://www.neuromorphometrics.org:8080/seg/html/segmentation/cerebral_white_matter.html). This atlas set will be referred to as the *MGC2012 database*.

**Figure 1:**
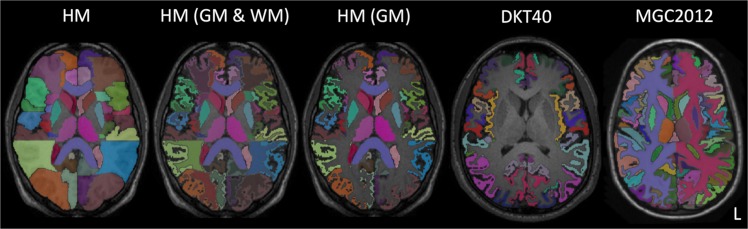
Axial cross-sections showing labels overlaid on the T1-weighted MRI of a sample subject from each of the atlas databases. Left to right: the unmodified HM atlas database, the HM atlas database with both grey-matter and white-matter sub-segmentations, the grey-matter masked HM atlas database, the DKT40 atlas database and the MGC2012 atlas database. Label colours are randomly assigned.

A cross-section of labels for an example subject in each atlas database is shown in Fig. 1. Atlas database MRI acquisition and participant details are given in Supplementary Table A1 and label names are listed in Supplementary Tables A2 to A4.

### Atlas properties

For each atlas set, we quantified inter-individual variation in region size across atlas subjects using the mean and standard deviation (SD) of region volumes, coefficient of variance (CV; defined as the standard deviation divided by the mean volume), and the surface-to-volume ratio (SVR). We compared each measure between atlas databases using a Kruskal-Wallis test for three samples, followed by Tukey-Kramer tests to identify significant differences between pairs of comparisons (Bonferroni-corrected for 3 comparisons). Region volumes for each subject were expressed as a fraction of intracranial volume (ICV) to account for inter-individual variations in ICV, multiplied by 10^4^ for ease of reading. The estimated ICV was obtained from FreeSurfer output (see Section *Segmentation method: FreeSurfer*). We investigated the influence of age on region volumes using Pearson’s correlations, sex differences in region volumes using two-tailed Student’s *t*-tests, and right-left differences in region volumes (excluding unpaired regions) using paired two-tailed *t*-tests, with Bonferroni correction applied to *p*-values for the number of regions in each atlas set (i.e. HM: *p* < 5.38 × 10^−4^; DKT40: *p* < 8.06 × 10^−4^; MGC2012: *p* < 3.68 × 10^−4^).

We also investigated the relationship between CV and SVR in each atlas database with two-tailed Pearson’s correlation coefficient tests, since the overlap measures used to measure segmentation accuracy are inherently sensitive to region volume and SVR, where the same level of inaccuracy in segmentation leads to a larger reduction in the overlap measure in regions with large SVRs^48^.

### Leave-one-out cross-comparison analysis

Each of the three atlas sets was used as the standard of reference for comparisons with automatically generated segmentation labels from FreeSurfer and MAPER (segmentation methods described in more detail below). For each atlas set, we conducted a leave-one-out cross-comparison analysis to estimate the accuracy with which the FreeSurfer and MAPER automatic segmentation methods can model the manual segmentation of a target image. Each subject’s MR image was treated as a test image in turn, with the remaining atlases (the training set) used to train the Gaussian classifier atlas (FreeSurfer) or used as label sources (MAPER).

### Segmentation method

#### FreeSurfer

Each subject’s T1-weighted MRI was first processed using the automated *recon-all* FreeSurfer processing stream (version 5.3.0; http://surfer.nmr.mgh.harvard.edu) to obtain the cortical surface reconstruction and tissue-class segmentation boundaries. No manual editing was performed to keep methods as automated as possible. Since FreeSurfer works in non-native space, the non-native atlases (HM and MGC2012 databases) needed to be resampled before they could be used as input atlases for FreeSurfer. The atlas labels were thus resampled using FreeSurfer’s *mri_vol2vol* tool with an identity matrix and nearest neighbour interpolation. Following *recon-all*, surface annotations of the volumetric atlas cortical labels were created using the FreeSurfer tool *mris_sample_parc* for each hemisphere. The left and right hemisphere surface annotations of all the training images were used to generate the Gaussian surface classifier (*mris_ca_train*) and subsequently label the test volume (*mris_ca_label*). Sub-cortical labels were combined with FreeSurfer reconstructions of the cortical grey and white matter labels to produce a modified aseg.mgz volume for each of the training images to generate the Gaussian classifier for sub-cortical regions (*mri_ca_train)* and produce sub-cortical labels for the test volume (*mri_ca_label*). The cortical and sub-cortical segmentations were then transferred into volumetric space using *mri_aparc2aseg*. The output segmentations were compared to the input atlases in FreeSurfer space to reduce the need for further resampling of the output segmentations.

Since FreeSurfer has separate streams for cortical and sub-cortical segmentations, we excluded regions in each non-native atlas that were split across the cortical and sub-cortical divisions as defined by FreeSurfer. These were the bilateral subcallosal area in the *HM database* and the bilateral basal forebrain in the *MGC2012 database*.

Tissue-classification results vary depending on the software used. To eliminate the effect of discordant grey matter definitions on the output of the segmentations, for the *HM database*, the FreeSurfer cortical grey matter mask was applied to both the original input atlases and the output segmentations.

#### MAPER

Each subject’s MRI was first processed using the standard MAPER pipeline (https://soundray.org/maper). Briefly, this involves reorienting each image to be segmented so it conforms to the FSL standard orientation, resampling to 1 mm^3^ isotropic voxels, field inhomogeneity correction^49^, brain extraction using pincram^50^, tissue-class segmentation using FSL FAST^45^, followed by pairwise registrations of each atlas-target combination. The brain masks, tissue-class segmentations, positional normalization parameters and multiple individually propagated atlas segmentations for the training set were used to generate the MAPER segmentation of the test volume.

For the *HM database*, results shown are from grey-matter masked labels applied to both input labels and output segmentations, based on the FSL FAST method described in the section *Atlas databases*.

### Comparison of segmentation methods

We compared the manual and automatic segmentation volumes using intraclass correlation coefficients (ICC, calculated using a two-way mixed effects model assessing absolute agreement in the ICC toolbox; https://uk.mathworks.com/matlabcentral/fileexchange/22099-intraclass-correlation-coefficient-icc) and limits of agreement using Bland-Altman plots. We report medians and interquartile ranges (IQR) for each atlas, and Wilcoxon rank-sum tests for differences between segmentation methods. To test for differences between atlas databases within segmentation methods, we used the Kruskal-Wallis Test for three samples, followed by Tukey-Kramer tests to identify significant differences between pairs of comparisons.

The accuracy of automatically generated labels was assessed by the amount of overlap with the target image segmentation per region, quantified using the Jaccard coefficient^51^ (JC; intersection divided by the union of the two labels). This translates into the commonly used, but less discriminating, Dice index^52^ (intersection divided by average) as $Dice=\frac{2\times JC}{1+JC}$.

Differences in segmentation accuracy between methods were assessed using two-tailed paired t-tests, with Bonferroni correction applied to p-values for the number of regions in each atlas set (see *Atlas properties* section for *p-*value thresholds).

As mentioned above, overlap measures, including JC, decrease with SVR and increase with region volumes. Low JC values are thus a weaker indicator of segmentation inaccuracy if the region is small or has a large SVR. To investigate this effect in each atlas database and for each segmentation method, we plotted JC against SVR and volume for each atlas set and computed Pearson’s correlation coefficients. Additionally, to compare JC values directly between atlas sets, we corrected JC for SVRs and region volumes within each segmentation method using linear regression.

### Validation on clinical datasets

Atlas sets are usually constructed using images of healthy participants but are often applied to investigate brain abnormalities in cohorts where such abnormalities are expected, e.g. cohorts of subjects with a certain disease. To investigate the performance of these methods and atlases in brains with pathological morphology, we applied the segmentation methods with each of the atlas databases to two cohorts consisting of patients with known structural abnormalities and their matched healthy control subjects.

The first clinical dataset consisted of MR images from the Alzheimer’s Disease Neuroimaging Initiative (ADNI) database (adni.loni.usc.edu, refer to ADNI website for details of ethical approval). We selected the 3 T T1-weighted MRI data acquired at baseline from patients with a diagnosis of AD and from healthy controls. Images and associated clinical data of 80 subjects in total were downloaded in April 2018. The sample consisted of 33 patients with AD (age range = 57–89 years; age mean ± SD = 74.0 ± 8.1; 22 female) and 47 healthy control subjects (age range = 70–86; age mean ± SD = 75.1 ± 3.9; 29 female).

The second clinical dataset consisted of MR images from patients with mesial temporal lobe epilepsy (mTLE) and unilateral hippocampal sclerosis (HS) who underwent preoperative MRI scanning, amygdalohippocampectomy, and postoperative follow-up at University Hospital Bonn, Germany. For each patient, HS was identified by an expert neuroradiologist with considerable experience of lesion diagnosis in epilepsy, and was defined by hippocampal volume loss and internal structure disruption on T1-weighted scans, and/or hyperintensities on T2-weighted and FLAIR images. No patient had evidence of bilateral HS or of a secondary extrahippocampal lesion that may have contributed to seizures. Histological confirmation of HS was performed using the standardized International League Against Epilepsy (ILAE) classification^53^. Images were obtained from the Life & Brain Center in Bonn, Germany on a 3 Tesla scanner (Magnetom Trio, Siemens, Erlangen, Germany). An eight-channel head coil was used for signal reception. Morphometric analyses in this study were performed on 3D T1-weighted MPRAGE images (160 slices, TR = 1300 ms, TI = 650 ms, TE = 3.97 ms, resolution 1.0 mm × 1.0 mm × 1.0 mm, flip angle 10°). A total of 177 subjects were included in the study, 41 with right HS (age range = 16–67; age mean ± SD = 41.0 ± 14.3; 17 female), 78 with left HS (age range = 17–70; age mean ± SD = 40.6 ± 13.3; 47 female) and 58 healthy control comparison subjects (age range = 18–67; age mean ± SD = 39.6 ± 13.4; 34 female). All patients and controls provided written informed consent, all methods were performed according to local ethics guidelines and regulations, and ethics approval was given by the Ethical Review Board of the Medical Faculty of Bonn.

The two clinical datasets were segmented using the segmentation methods and atlas databases described above. For each combination of segmentation method and atlas database, we compared structure volumes between each patient group (AD, left-HS and right-HS) and their healthy control groups using multivariate analysis of covariance (ANCOVA), with age, sex and ICV as covariates and Bonferroni correction for the number of regions in each atlas (see *Atlas properties* section for *p-*value thresholds). ICV was estimated from the FreeSurfer output and used in the ANCOVA for both segmentation methods.

## Results

### Atlas properties

Atlas properties are summarised in Table 1. After accounting for ICVs, post-hoc tests showed that CVs were significantly higher in the MGC2012 atlas compared to both HM (*p* = 0.005) and DKT40 (*p* = 0.009), and SVRs were significantly different between all pairs of atlas databases (HM vs. DKT40: *p* = 0.004; MGC2012 vs. DKT40 & HM: both *p* < 0.001). Detailed region statistics for each atlas set are given in Supplementary Tables B1 to B3.

**Table 1:** Atlas region properties.

	HM	DKT40	MGC2012
**Region Volumes**^**†**^			
Range	2.08–461	7.00–176	0.57–1448
Mean ± SD	48.3 ± 74.6	52.3 ± 36.2	63.9 ± 177
**Region CV**^**‡**^			
Range	0.06–0.47	0.10–0.40	0.08–0.73
Mean ± SD	0.19 ± 0.08	0.19 ± 0.07	0.25 ± 0.13
**Region SVR**^**§**^			
Range (Max/Min)	0.20–1.80 (9.0)	0.58–1.24 (2.1)	0.32–1.85 (5.8)
Mean ± SD	1.12 ± 0.31	0.94 ± 0.12	0.78 ± 0.23

^†^volumes expressed as a fraction of ICV × 10^4^. ^‡^CV: coefficient of variance (standard deviation divided by mean). ^§^SVR: surface area to volume ratio.

There were no significant sex differences in region volumes in any of the three atlas databases. In the MGC2012 atlas, significant correlations with age were found in 5/134 regions. All five were ventricular regions and had positive correlations with age: third ventricle (r = 0.79), right inferior lateral ventricle (r = 0.71), left inferior lateral ventricle (r = 0.68), right lateral ventricle (r = 0.74) and left lateral ventricle (r = 0.76). No significant correlations of region volumes with age were found in the HM or DKT40 atlas databases.

In the HM database, significant right-left differences were found in 5/46 paired structures. Regions larger on the left were the nucleus accumbens (7.0%), putamen (2.2%) and thalamus (1.4%). The hippocampus (3.6%) and temporal horn of the lateral ventricle (9.0%) were larger on the right. Significant right-left differences were found in 3/31 structures in the DKT40 database, with the superior temporal gyrus (4.0%) and transverse temporal gyrus (9.2%) larger on the left, and the pericalcarine cortex (5.4%) larger on the right. Significant right-left differences were found in 4/64 paired structures in the MGC2012 database, with the lateral ventricle (8.9%), thalamus (2.3%) and ventral diencephalon (2.6%) larger on the left, and the hippocampus (2.8%) larger on the right.

**Figure 2:**
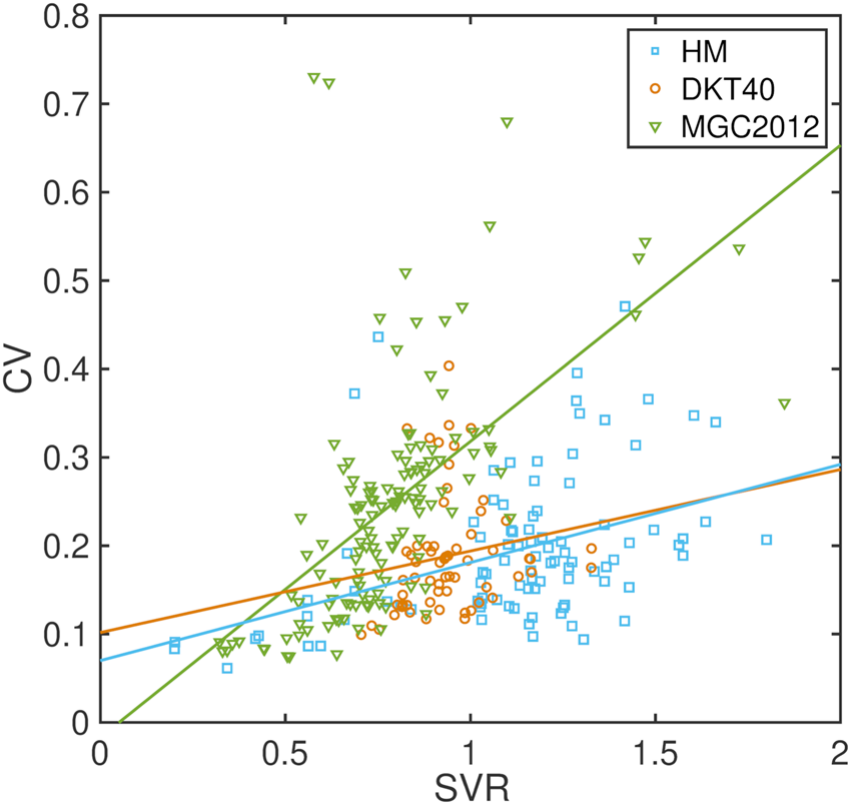
Plot of CV vs SVR with lines of least squares fit for each atlas database.

Figure 2 shows significant positive correlations between SVR and CV in two of the three atlas databases (HM: r = 0.41, p < 0.001; DKT40: r = 0.17, p = 0.188; MGC2012: r = 0.59, p < 0.001). In general, across all atlas sets, higher SVRs led to higher CVs, in line with previously reported findings^48^, but this effect was more pronounced in the MGC2012 database.

### Leave-one-out cross-comparison analysis

#### Manual vs. automatic segmentation volumes

**Figure 3:**
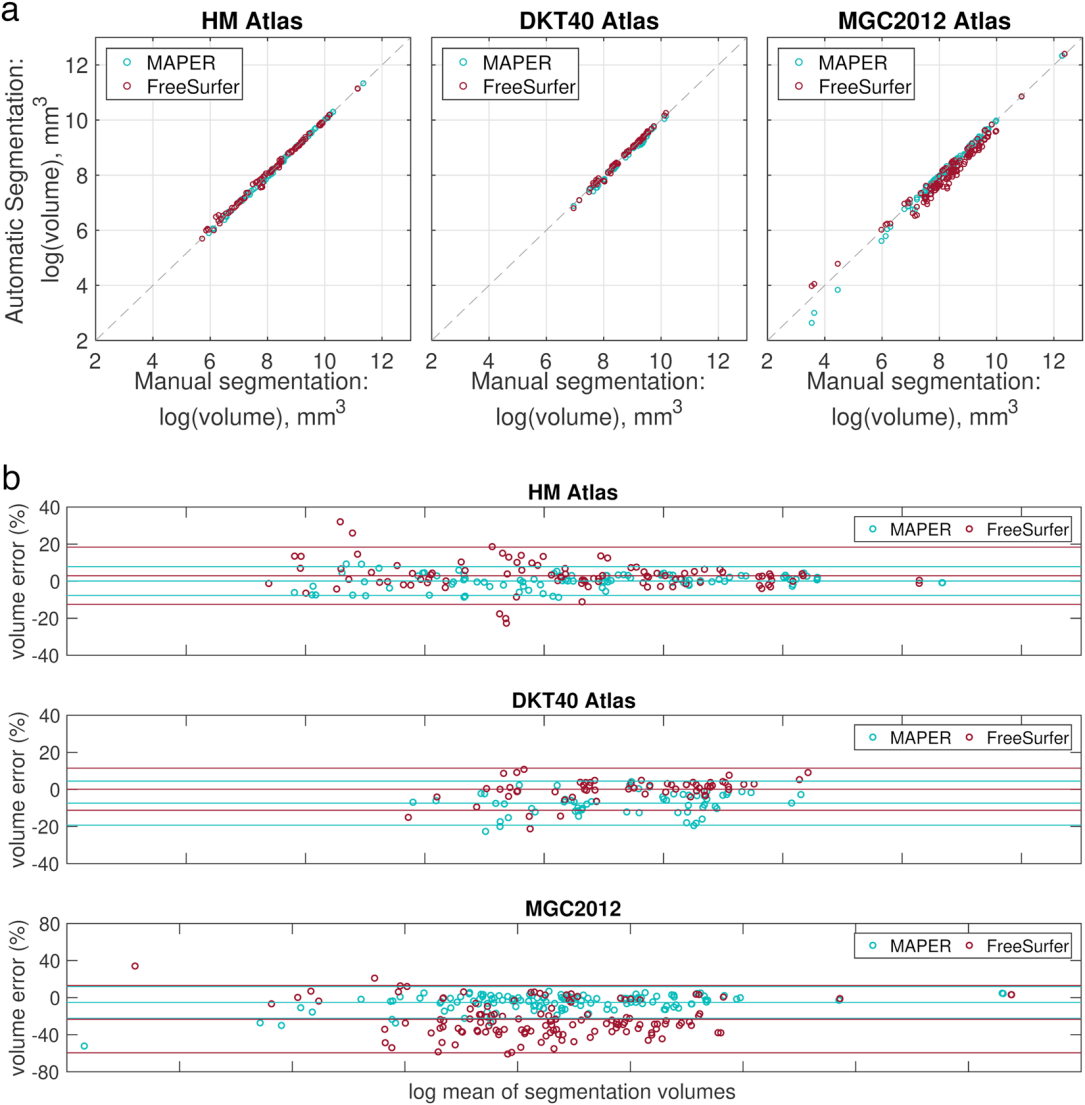
Comparisons of manual vs. automatic segmentation volumes. (**a**) Plots of manual vs. automatic segmentation volumes for all subjects. Volumes are in mm^3^. The grey dashed line denotes x = y. (**b**) Bland-Altman plots for comparison between log transformed mean region volumes (mm^3^) of manual and automatic segmentations, and the volume error between automatic and manual segmentations across all regions in each atlas set. The lines show the mean and 95% confidence intervals. Volumes are in mm^3^. Note the different ranges on the y axes.

ICCs were significantly different between segmentation methods for all atlas sets (Fig. 3a and Table 2; all *p* < 0.001). MAPER with the HM atlas had the highest median correlation with manually segmented region volumes, while FreeSurfer with the MGC2012 atlas had the lowest. Comparing between atlas databases for the MAPER segmentation method, the HM atlas database had significantly higher ICCs than both the DKT40 and MGC2012 databases (both *p* < 0.001, follow-up Tukey-Kramer tests). For the FreeSurfer segmentation method, there was no significant difference between HM and DKT40, and significantly lower ICCs for the MGC2012 compared to both the HM and DKT40 atlas databases (both *p* < 0.001, follow-up Tukey-Kramer tests).

**Table 2:** ICCs, volume errors and Jaccard overlaps between manual and automatic segmentation volumes.

	MAPER	FreeSurfer
**Volume ICC (median ± IQR)**		
HM	0.83 ± 0.22	0.69 ± 0.31
DKT40	0.65 ± 0.22	0.80 ± 0.21
MGC2012	0.64 ± 0.35	0.38 ± 0.45
**Volume Error % (median ± IQR)**		
HM	0.49 ± 4.90	2.45 ± 7.24
DKT40	−6.69 ± 8.76	1.14 ± 4.57
MGC2012	−2.77 ± 9.10	−27.1 ± 30.1
**Original JCs (mean ± SD)**		
HM	0.73 ± 0.09	0.68 ± 0.11
DKT40	0.61 ± 0.06	0.72 ± 0.06
MGC2012	0.62 ± 0.12	0.52 ± 0.14
***Corrected JCs (mean*** **±** ***SD)***		
HM	0.74 ± 0.06	0.67 ± 0.08
DKT40	0.60 ± 0.05	0.69 ± 0.06
MGC2012	0.62 ± 0.10	0.54 ± 0.13

IQR denotes interquartile range.

We define the volume error between manual and automatic segmentations as:

$$\frac{vo{l}_{m}-vo{l}_{a}}{\frac{1}{2}\times (vo{l}_{m}+vo{l}_{a})}\times 100 \% $$

where *vol*_*m*_ is the manual segmentation volume and vol_a_ is the automatic segmentation volume. Figure 3b shows Bland-Altman plots of volume errors against the log mean of segmentation volumes. The median volume error was smallest for the HM atlas using the MAPER segmentation method (Table 2). There were significant differences in volume error between segmentation methods for all atlas sets. Using the DKT40 atlas, MAPER tended to underestimate structure volumes while FreeSurfer tended to overestimate structure volumes. The volume error was largest for the MGC2012 atlas using the FreeSurfer segmentation method, where FreeSurfer tended to underestimate cortical structure volumes. Comparing between atlas databases for the MAPER segmentation method, the HM atlas database had significantly smaller volume errors than both the DKT40 and MGC2012 databases. For the FreeSurfer segmentation method, there was no significant difference between HM and DKT40, and significantly larger volume errors for the MGC2012 compared to both the HM and DKT40 atlas databases.

#### Differences in automatic-to-manual label agreement

**Figure 4:**
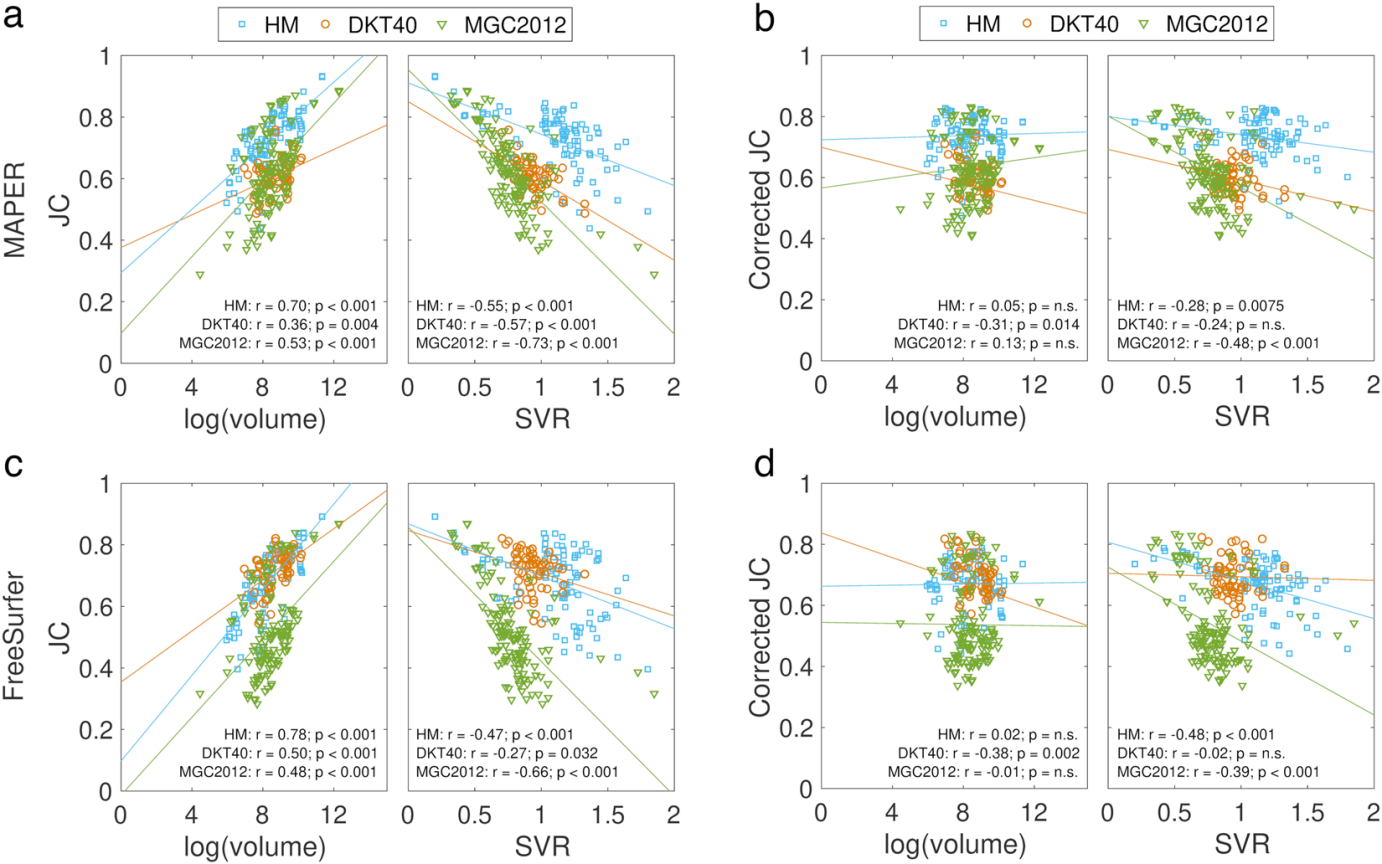
Plots of JC vs log(volume) and JC vs SVR with lines of least squares fit in each atlas set and for each segmentation method. (**a**) Raw JC values for MAPER segmentation, (**b**) JC values after correcting for SVR and log(volume) for MAPER segmentation, (**c**) raw JC values for FreeSurfer segmentation, (**d**) corrected JC values for FreeSurfer segmentation.

JCs were negatively correlated with SVRs and positively correlated with region volume in all three atlas databases and both segmentation methods (Fig. 4). To be able to compare JCs directly between atlas databases, we corrected JC for region volume and SVR using linear regression. We show both the original and corrected mean JCs in Table 2. MAPER with the HM atlas had the highest mean JC of all atlas database and segmentation method combinations and the HM atlas performed best regardless of segmentation method (all follow-up tests *p* < 0.001).

**Figure 5:**
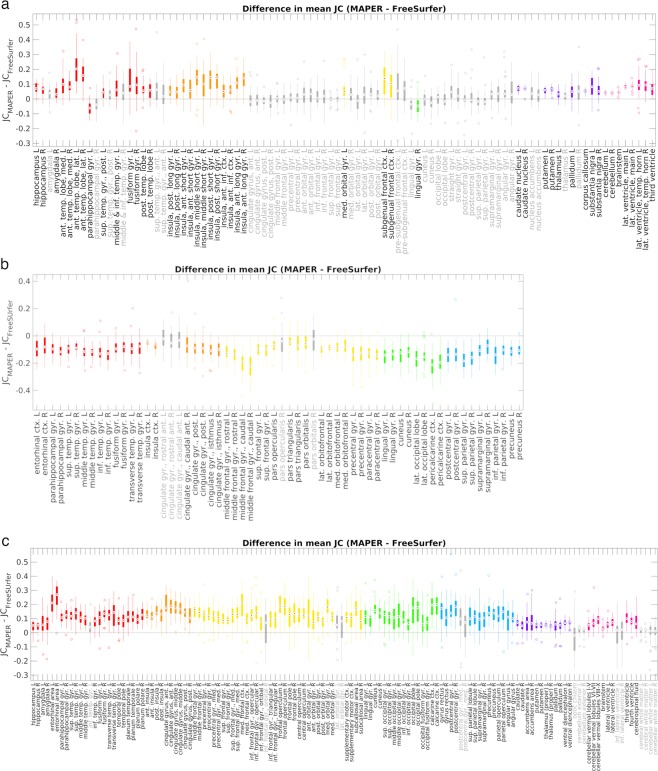
Boxplot of JC differences between segmentation methods for each atlas database. Positive differences indicate higher mean JC for MAPER than FreeSurfer. Significant differences in JC (from Welch’s two-tailed paired t-test, after adjustment for multiple comparisons) are indicated by coloured boxes and black region labels on the x-axis. Non-significant JC differences are shown in grey. Regions are colour coded by lobe: red – temporal lobe, orange – insula & cingulate, yellow – frontal lobe, green – occipital lobe, blue – parietal lobe, purple – central structures, pink – posterior fossa & ventricles. The top and bottom edges of the boxes are the 25^th^ and 75^th^ percentiles, the target inside each box is the median and outliers are indicated by unfilled circles on either end of the box whiskers. Abbreviations: L = left, R = right, G = gyrus, ctx = cortex, ant. = anterior, pos. = posterior, sup. = superior, inf. = inferior, med. = medial, lat. = lateral, temp. = temporal. (**a**) HM atlas, (**b**) DKT40 atlas, (**c**) MGC2012 atlas.

Differences in label agreement between MAPER and FreeSurfer for each of the 93 labels in the HM atlas from the leave-one-out cross-comparisons are shown in Fig. 5a. The overall mean JC across all subjects and regions was significantly higher for MAPER than for FreeSurfer (t(92) = 9.37, *p* < 0.001). MAPER had significantly larger overlaps for 45 of the 93 labels primarily in the temporal lobes, insula and sub-cortical regions, while FreeSurfer had significantly larger overlaps for two labels, the left parahippocampal gyrus and the right lingual gyrus.

Differences in label agreement between methods for each of the 62 cortical labels in the DKT40 atlas are shown in Fig. 5b. The overall mean JC across all subjects and regions was significantly higher for FreeSurfer than for MAPER (t(61) = 19.2, *p* < 0.001). FreeSurfer had significantly higher JC for 56 of the 62 regions while MAPER did not have any regions that showed significantly higher JC.

Differences in label agreement between methods for each of the 132 cortical and sub-cortical labels in the MGC2012 atlas are shown in Fig. 5c. The overall mean JC across all subjects and regions was significantly higher for MAPER than for FreeSurfer (t(131) = 24.1, *p* < 0.001). MAPER had significantly higher JC for 118 of the 132 labels while FreeSurfer had no labels with significantly higher JC than MAPER.

Per region overlaps for each atlas are detailed in Supplementary Tables C1 to C3 and boxplots of label agreement across all subjects for each region and segmentation method are shown in Supplementary Figs. S1 to S3.

### Validation on clinical datasets

#### Alzheimer disease data (ADNI)

A summary of the top five largest group differences for each segmentation method and atlas set is shown in Table 3, and further details are given in Supplementary Tables D1 to D3.

**Table 3:** Top five group differences between healthy control (HC) subjects and patients with Alzheimer’s Disease (AD).

MAPER			FreeSurfer		
Structure	% diff	p-value	Structure	% diff	p-value
**HM Atlas**					
hippocampus L	−22.68	9.44E-11	hippocampus L	−25.59	7.31E-12
hippocampus R	−17.69	1.46E-08	amygdala L	−25.78	3.71E-09
parahippocampal G L	−17.31	3.39E-08	parahippocampal G L	−23.12	9.95E-09
lat. ventricle, main R	37.03	2.16E-06	parahippocampal G R	−22.52	1.04E-08
parahippocampal G R	−14.05	6.53E-06	hippocampus R	−20.67	1.98E-08
**DKT40 Atlas**					
parahippocampal G L	−18.94	1.20E-06	entorhinal cort. L	−27.03	5.32E-09
entorhinal cort. L	−19.84	3.65E-06	entorhinal cort. R	−26.09	1.70E-07
entorhinal cort. R	−17.28	3.12E-05	parahippocampal G R	−15.93	4.35E-06
cingulate G, isthmus L	−15.51	6.28E-05	fusiform G R	−11.60	4.63E-05
middle temp. G L	−9.80	1.09E-04	cingulate G, isthmus L	−12.36	1.33E-04
**MGC2012 Atlas**					
lateral ventricle R	46.22	1.36E-06	hippocampus L	−22.88	2.54E-11
amygdala L	−18.24	3.07E-06	hippocampus R	−20.00	4.16E-10
parahippocampal G L	−13.42	5.99E-06	parahippocampal G R	−20.79	1.42E-08
lateral ventricle L	47.44	7.03E-06	parahippocampal G L	−19.88	2.68E-08
amygdala R	−17.04	9.45E-06	amygdala L	−37.26	4.86E-08

Regions arranged by p-value. Negative percentage difference values indicate smaller volumes in AD than HC. All comparisons were significantly different between groups after Bonferroni correction (HM: *p* < 5.38 × 10^−4^; DKT40: *p* < 8.06 × 10^−4^; MGC2012: *p* < 3.68 × 10^−4^). L: left, R: right, G: gyrus, lat.: lateral, cort.: cortex, temp.: temporal. Note the DKT40 atlas does not contain a hippocampus region.

The number of regions that were significantly different between patients with AD and healthy controls were as follows: HM with FreeSurfer – 12/93; HM with MAPER – 14/93 structures; DKT40 with FreeSurfer – 11/62; DKT40 with MAPER – 9/62 structures; MGC2012 with FreeSurfer – 16/132; MGC2012 with MAPER – 15/132 structures. For all atlases, FreeSurfer generally showed an overall larger percentage difference in region volumes between groups and lower p-values for the comparisons. All regions in all comparisons have biological plausibility; note that the DKT40 atlas does not contain hippocampi.

#### Hippocampal sclerosis data

The top five largest group differences for each comparison are shown in Tables 4 and 5, for left and right HS compared to controls respectively, and further details are given in Supplementary Tables E1 to E6.

**Table 4:** Top 5 group differences between healthy control subjects and patients with left hippocampal sclerosis.

MAPER			FreeSurfer		
Structure	% diff	p-value	Structure	% diff	p-value
**HM Atlas**					
**hippocampus L**	**−32.52**	**7.00E-20**	**hippocampus L**	**−34.15**	**8.00E-20**
**third ventricle**	**21.87**	**4.03E-04**	**sup. temp. G, ant. L**	**−16.82**	**1.14E-06**
thalamus L	−8.17	7.10E-04	**thalamus L**	**−9.39**	**1.80E-06**
substantia nigra L	−8.32	1.77E-03	**ant. temp. lobe, med. L**	**−15.64**	**2.54E-05**
sup. frontal G R	−7.19	1.90E-03	**postcentral G R**	**−10.59**	**2.70E-05**
**DKT40 Atlas**					
entorhinal cort. L	−8.91	5.26E-03	**sup. temp. G L**	**−12.22**	**2.64E-06**
middle temp. G L	−6.16	5.59E-03	**postcentral G R**	**−11.89**	**3.79E-05**
postcentral G R	−5.81	5.79E-03	**middle frontal G, rostral L**	**−9.95**	**2.21E-04**
parahippocampal G R	6.41	1.72E-02	inf. temp. G L	−8.29	9.68E-04
sup. temp. G L	−3.40	2.89E-02	entorhinal cort. L	−14.62	1.04E-03
**MGC2012 Atlas**					
**hippocampus L**	**−23.01**	**1.95E-17**	**hippocampus L**	**−29.67**	**4.35E-21**
**thalamus (proper) L**	**−10.23**	**1.67E-05**	**temporal pole L**	**−16.85**	**4.07E-07**
**third ventricle**	**26.03**	**1.90E-04**	**parahippocampal G L**	**−11.62**	**2.88E-05**
cerebral white matter L	−4.55	4.29E-04	**postcentral G R**	**−13.03**	**3.69E-05**
parahippocampal G R	6.94	1.63E-03	**thalamus (proper) L**	**−7.12**	**1.22E-04**

Regions arranged by p-value. Negative percentage difference values indicate smaller volumes in patients than controls. Entries in bold were regions that were significantly different between groups after Bonferroni correction (HM: *p* < 5.38 × 10^−4^; DKT40: *p* < 8.06 × 10^−4^; MGC2012: *p* < 3.68 × 10^−4^). L: left, R: right, sup.: superior, temp.: temporal, ant.: anterior, med.: medial, G: gyrus, cort.: cortex, inf.: inferior. Note the DKT40 atlas does not contain a hippocampus region.

**Table 5:** Top five group differences between healthy control subjects and patients with right hippocampal sclerosis.

MAPER			FreeSurfer		
Structure	% diff	p-value	Structure	% diff	p-value
**HM Atlas**					
**hippocampus R**	**−36.23**	**1.67E-15**	**hippocampus R**	**−38.12**	**6.70E-12**
fusiform G L	8.78	3.50E-03	**thalamus R**	**−9.23**	**1.41E-05**
thalamus R	−6.52	4.94E-03	postcentral G R	−9.81	5.67E-04
insula, middle short G R	−13.57	7.86E-03	ant. temp. lobe, med. R	−11.91	7.52E-04
parahippocampal G L	6.79	1.20E-02	sup. temp. G, ant. R	−13.89	1.10E-03
**DKT40 Atlas**					
parahippocampal G R	−9.99	1.62E-02	**postcentral G R**	**−11.13**	**6.09E-04**
middle temp. G R	−6.01	1.88E-02	sup. temp. G R	−6.83	1.31E-02
entorhinal cort. L	8.85	4.39E-02	precentral G R	−7.17	2.11E-02
pars triangularis L	5.93	5.30E-02	inf. parietal G L	−6.10	2.87E-02
precuneus R	−4.32	7.29E-02	precentral G L	−6.27	2.96E-02
**MGC2012 Atlas**					
**hippocampus R**	**−27.25**	**3.58E-15**	**hippocampus R**	**−38.33**	**4.89E-13**
**thalamus (proper) R**	**−9.21**	**2.34E-04**	**thalamus (proper) R**	**−8.18**	**2.21E-05**
amygdala L	9.39	2.97E-03	precentral G R	−12.66	4.39E-04
parahippocampal G L	6.15	6.02E-03	temporal pole R	−10.49	2.01E-03
cerebral white matter R	−3.84	6.44E-03	parietal operculum R	11.87	2.29E-03

Regions arranged by p-value. Negative percentage difference values indicate smaller volumes in patients than controls. Entries in bold were regions that were significantly different between groups after Bonferroni correction (HM: *p* < 5.38 × 10^−4^; DKT40: *p* < 8.06 × 10^−4^; MGC2012: *p* < 3.68 × 10^−4^). L: left, R: right, G: gyrus, med.: medial, ant.: anterior, temp.: temporal, sup.: superior, cort.: cortex, inf.: inferior. Note the DKT40 atlas does not contain a hippocampus region.

As expected, there were far fewer regions of significant differences than for the patients with AD: using the HM atlas, FreeSurfer showed significant differences between patients with HS and healthy controls in 7/93 regions versus 2/93 for MAPER for patients with left HS; for patients with right HS, there were 2/93 significantly different regions using FreeSurfer and 1/93 using MAPER. Using the DKT40 atlas, the number of regions with significant differences for FreeSurfer were 3/62 for left HS and 1/62 for right HS, whereas there were no regions with significantly different volumes between the patients and controls using MAPER (note that the DKT40 atlas does not contain a hippocampus region). Using the MGC2012 atlas, FreeSurfer found significantly different volumes from controls in 6/132 regions for patients with left HS and 2/132 regions for patients with right HS; for MAPER, these numbers were 3/132 regions for left HS and 2/132 regions for right HS. The region of maximal differences was plausible, i.e. the ipsilateral hippocampus, for both methods and both atlases that contain a hippocampus region.

## Discussion

In this study, we present a comprehensive evaluation of two brain segmentation methods using three atlas databases. We present detailed descriptive data comparing three commonly used atlas databases and show that the databases differ in quality.

Both segmentation methods reliably identify known abnormalities in each patient group; FreeSurfer separated better between patients and healthy controls in the AD and left HS datasets, whereas MAPER performed better for the right HS dataset.

CVs for region volumes for the HM and DKT40 atlas databases were similar, while the MGC2012 atlas showed a higher mean CV across all regions. The HM atlas database had the largest range of SVR (max/min = 9.0), followed by MGC2012 (5.8), and DKT40 (2.1). This has implications in interpreting the overlap between automatic and manual segmentations, because overlap tends to decrease in regions with higher SVR and smaller volumes. The MGC2012 atlas database shows a stronger correlation between regional CVs and SVRs than the other two databases, indicating a more heterogeneous spread of volumes in this atlas compared to regions of similar shape in the other two atlases, consistent with the larger age range but possibly also suggesting lower consistency of manual segmentations.

The MGC2012 atlas database showed some regional volume correlations with age, while the HM and DKT40 atlas databases did not. This might be expected because of a larger age range and variation in the MGC2012 database subjects. Correlations with age in the MGC2012 atlas database are largely concordant with what is known from the literature, i.e. reduction in volumes of caudate and frontal gyri, and increase in ventricular volumes with age^54^.

In all three atlas databases, right-left asymmetry in brain volumes was also largely concordant with known differences in healthy adults: for example, regions larger on the left include the accumbens and thalamus^54,55^ and regions larger on the right include the hippocampus^56^ and pericalcarine cortex^57^.

Another important distinction between atlas databases is the presence of detailed white matter labels. The HM atlas has labels that encompass both grey and white matter for each cortical region, and hence detailed white matter labels can be obtained by using a white-matter mask derived with any of the standard neuroimaging software packages. A limitation of white-matter labels generated in this fashion is that boundaries between them are conditioned on features of the cortex, rather than on intensity gradients or other image features local to these boundaries. Still, for certain diseases or applications, such detailed white-matter segmentations may be of interest.

Some attempts at quantifying differences in atlases have been made previously^58,59^, and it has been shown that there is an overall lack of agreement in region boundaries and definitions between atlas databases. Differences lie not only in the protocols for outlining brain regions and the number of brain regions available, but also in the sample of subjects included in the database. A fair assessment of the quality of atlas databases is not easy to achieve, since several factors contribute to regional variance, and it is not always easy or possible to distinguish these effects. Factors affecting variation between atlases include inter-individual brain differences (e.g. related to age), the quality and consistency of expert delineations (i.e. inter- and intra-rater reliability), the ease of delineation of regions (e.g. some regions have more inconsistent boundaries, while others are less variable between individuals), and the surface-to-volume ratio of regions^22^. It is useful for users to be aware of the characteristics of these atlas databases, as the choice of atlas has implications on reproducibility of regions and suitability for use with different segmentation methods.

Volumetric comparisons between manual and automatically generated volumes revealed overall better segmentation accuracy for MAPER than for FreeSurfer in the HM and MGC2012 atlas databases, while FreeSurfer had smaller volume errors than MAPER for the DKT40 atlas. As expected, both segmentation methods performed better using their native atlas databases. According to FreeSurfer documentation (https://surfer.nmr.mgh.harvard.edu/fswiki/FreeSurferBeginnersGuide), FreeSurfer requires high contrast between grey and white matter in order to perform well. FreeSurfer segmentation quality may change with image quality and the results may conceivably change when using MRIs acquired with different settings or from different field strengths. Also, FreeSurfer processing requires image resampling, rather than working in native space. While we have tried to minimise the impact of interpolation by resampling only the input atlases, then comparing the output segmentations (already in FreeSurfer space) to the resampled input atlases, information loss incurred during the initial resampling may have an impact on the results.

Overlap comparisons between manual and automatically generated volumes produced similar results, with MAPER producing higher JC values than FreeSurfer for the HM and MGC2012 atlas databases, and FreeSurfer producing higher JCs than MAPER for the DKT40 atlas database. Both segmentation methods performed worse with the MGC2012 atlas, and the JC vs. SVR plots showed a steeper decline in JC with increased SVR in the MGC2012 atlas. This difference may be related to the variability of regions in the MGC2012 atlas (c.f. higher CVs with higher SVRs in MGC2012).

Overall, the HM atlas database performed best in terms of consistency of automatic segmentation of healthy controls including across segmentation methods, and stability of variation across SVRs. Regardless of the segmentation method used, manual labels in the HM atlas database were reproduced with higher fidelity than those in the DKT40, which in turn was better than the MGC2012 atlas. This effect was seen in both the comparison of ICCs and JCs between manual and automatic labels – the HM atlas had the highest ICCs and the highest leave-one-out Jaccard overlap averaged between the MAPER and FreeSurfer segmentation methods at 0.71 compared with 0.65 for DKT40 and 0.58 for MGC2012, and the smallest difference between the two segmentation methods. As ICCs are high when intrasubject variability is small but inter-subject variability is large, this indicates that the effect is not due to labels lacking complexity, a finding additionally supported by the average SVR which is highest for the HM atlas database. This suggests that the HM database has the highest quality of the three databases under consideration in this study.

Segmenting imaged cohorts of patients and controls enables region-by-region volumetric group comparisons that can reveal neuroanatomical correlates of the disease state. Known disease correlates will be seen more or less distinctly, depending on the validity of the segmentation method applied. Studying disease cohorts thus offers the opportunity to compare segmentation methods in a fashion that is tied to a realistic application scenario. A segmentation method may be regarded as superior to another if it shows the difference between a diseased brain and a healthy brain more distinctly.

In the patients with AD, we found that regions identified as most significantly different from controls in all three atlas sets, and segmentation methods all have biological plausibility and are consistent with known abnormalities in AD: bilateral atrophy of the hippocampi, parahippocampal gyri, and amygdalae, along with enlargement of the lateral ventricles^60,61^. In the DKT40 atlas, regions showing atrophy included the bilateral entorhinal cortex, left middle temporal gyrus, and left isthmus of the cingulate gyrus (note that the hippocampus and amygdala are not available with this atlas). The results were remarkably similar in the HM and MGC2012 atlases with both segmentation methods, although the HM atlas with FreeSurfer was best able to distinguish between groups based on *p*-values. Comparing between atlas databases only, the HM atlas database showed overall lower *p*-values regardless of segmentation method. Comparing between segmentation methods, FreeSurfer was better able to distinguish between groups regardless of atlas database. It is worth noting that the larger age range in the MGC2012 atlas did not give it an advantage in segmenting the AD cohort with higher age ranges.

It may seem paradoxical that FreeSurfer performs better at separating patient and healthy control groups based on brain volumes, even though MAPER outperforms FreeSurfer in the manual vs. automatic segmentation analysis within the healthy controls. One explanation for this is that FreeSurfer overestimates region volumes, especially in larger brains, thus enhancing atrophy-related discrepancies. As an example, the mean volume of the left hippocampus in healthy controls was larger in the FreeSurfer segmentation of the HM atlas than the MAPER segmentation (1921 mm^3^ vs 1841 mm^3^), and the mean volume in AD was approximately the same in the FreeSurfer and MAPER segmentations (1428 mm^3^ and 1423 mm^3^ respectively). While these individual differences in volume were not significant between the two segmentation methods, they contributed to an overall greater difference between the patient and control groups in FreeSurfer volumes. The overestimation in larger brains found here was also reported in two other studies comparing automatic hippocampal segmentation methods using FreeSurfer, which additionally found underestimation in smaller brains^12,62^.

In the HS dataset, the most significant difference between HS patients and healthy controls was low volume of the affected (ipsilateral) hippocampus in HS patients, which was expected, given that hippocampal atrophy is the defining feature of HS. Both segmentation methods, when combined with atlas databases containing a hippocampus region (i.e. the HM and MGC2012 atlas databases), were able to identify ipsilateral hippocampal atrophy in HS patients. FreeSurfer with the MGC2012 atlas gave the largest and most significant difference between left HS patients and controls in the left hippocampus, whereas MAPER combined with the HM atlas showed the most significant difference, although not the largest percentage atrophy, between right HS patients and controls in the right hippocampus. Outside of the affected hippocampus, there was less concordance of results across segmentation methods and atlas databases. Other regions showing abnormality in HS patients include the thalamus on the affected side and temporal lobe regions, although the results are more mixed. Atrophy in the thalamus is one of the more widely reported extra-temporal abnormalities found in mTLE patients^63^. A peculiar result was the smaller volume of the right postcentral gyrus in both left and right HS patients found with FreeSurfer and the DKT40 atlas, and with FreeSurfer combined with the two other atlases in left HS patients. On qualitative inspection, MR images do not appear to show any consistent difference between HS patients and controls in the right postcentral gyrus. It is interesting that a similar finding, albeit for cortical thickness and not volume, has recently been reported in a large meta-analysis using FreeSurfer^64^. Further investigation is warranted into whether this unexpected finding is due to biology or methodology, considering the special challenge of this region of the brain where the cortex is particularly thin.

Our analyses highlight the importance of atlas choice and segmentation method. This may be particularly important when abnormalities are focal rather than affecting the whole brain. For example, in HS patients, there is a robust abnormality in the affected hippocampus, but extra-hippocampal abnormalities are subtler or not present in all patients^65^. This also has implications in investigating atrophy in subjects with subtle and heterogeneous abnormalities, for example patients with mild cognitive impairment.

We applied the methods without manual intervention, even though FreeSurfer explicitly invites this. Tissue-class segmentation and atlas-based segmentation are often packaged together within the same segmentation software, but it is difficult to disentangle tissue-class segmentation from region segmentation in the above methods because each segmentation method uses its own tissue-class segmentation. To complicate things further, the MGC2012 atlas database has explicitly labelled white matter. This difference in tissue-class definitions could explain the underestimation in volumes by FreeSurfer (c.f. Fig. 3b), with the FreeSurfer cortical ribbon being estimated as thinner than what was labelled with the MGC2012 atlas. This difference in tissue-class segmentation also makes it difficult to compare between surface- and volume-based segmentation methods.

Other segmentation options include patch-based segmentation, recently expanded to enable multi-region segmentations^66^, and deep learning methods^67–69^. Deep learning methods offer the promise of rapid segmentation once time-consuming training has been performed, but have not always achieved the accuracy of multi-atlas or patch-based methods in formal comparisons^70,71^. They are susceptible to overfitting to a particular training set and often do not transfer across different image acquisition sequences and MRI scanners^72^. Deep learning methods perform best with large numbers of training datasets; careful evaluation of the quality of the reference, as undertaken in this work, will remain a prerequisite.

Although a large number of atlases and parcellation schemes exist for the brain, we only used three atlas databases in this study, focussing on atlases that are freely available and have expert manual delineations of the whole brain for multiple subjects. More recently, new parcellation schemes that take advantage of multiple modalities (structural, functional connectivity, gene expression, etc.) have been developed^30^. While these are more likely to present a complementary picture of brain structural and functional organisation, they were not considered within the scope of this study because of the nature of the comparisons here that require manual labels based on T1-weighted structural imaging. Different applications will likely use different types of atlases.

This evaluation was designed with a view to providing some guidance on the choice of atlas and segmentation methods. The demands of the application and the user’s priorities determine which combination is optimal. Unsurprisingly, segmentation methods tend to perform best when using the native atlases with which they were developed. Users providing their own atlases are cautioned about the potentially lower quality of automatic segmentations produced when using a non-native atlas.

Our results suggest that automatic segmentation using MAPER produces labels closer to manual segmentations in healthy controls, but FreeSurfer performs better at distinguishing between patient cohorts and healthy controls. Both methods perform well at identifying correlates of disease when the discrepancy versus controls is large, but atlas choice and segmentation method matter more when abnormalities are subtler. This is a particularly important consideration when comparing results from studies using different methods.

We also show that available atlas resources differ with regard to the quality of the manual segmentations, with the HM atlas showing superior results in the majority of comparisons.

The results shown here are a useful guide for the expected accuracy, and thus the interpretation of results from analyses, of segmentations depending on region size, SVRs and segmentation method. The findings from this study will also inform the further development of the MAPER software and Hammers_mith atlas database.

## Supplementary information

Supplementary Material.

## Data Availability

The segmentations generated in this study using MAPER and FreeSurfer for each atlas database are available to download from https://osf.io/pv39g/. The MAPER and pincram software are available on GitHub: https://github.com/soundray/maper; https://github.com/soundray/pincram. FreeSurfer can be downloaded from http://www.freesurfer.net. All atlas data used in this study were downloaded from their respective websites: the Hammers_mith atlases from http://brain-development.org/brain-atlases/adult-brain-atlases, the Desikan-Killiany-Tourville atlases from https://mindboggle.info/data.html, and data can be requested for the MICCAI 2012 Grand Challenge and Workshop on Multi-Atlas Labelling atlases from https://my.vanderbilt.edu/masi/workshops. ADNI data can be requested from http://adni.loni.usc.edu. TLE data can be made available by Bernd Weber on reasonable request.
