# Supplementary material for: On brain atlas choice and automatic segmentation methods: a comparison of MAPER & FreeSurfer using three atlas databases
Source: Sci Rep. 2020 Feb 18;10:2837. doi: 10.1038/s41598-020-57951-6 (PMC7028906; doi:10.1038/s41598-020-57951-6)

Supplementary Material

On brain atlas choice and automatic segmentation methods: a comparison of MAPER & FreeSurfer using three atlas databases

S.N. Yaakub, R.A. Heckemann, S.S. Keller, C.J. McGinnity, B. Weber, A. Hammers

METHODS

Supplementary Methods: Masking to achieve compatibility between HM and the other atlases.........2

TABLES

Atlas database details

Supplementary Table A1. Summary of atlas databases. 2
Supplementary Table A2. List of 95 cortical and sub-cortical labels for HM atlas. 3
Supplementary Table A3. List of 62 cortical labels for DKT40 atlas. 4
Supplementary Table A4. List of 132 cortical and sub-cortical labels for MGC2012 atlas. 5

Summary statistics of atlas properties

Supplementary Table B1. Region properties in HM atlas database. 6
Supplementary Table B2. Region properties in DKT atlas database. 8
Supplementary Table B3. Region properties in MGC2012 atlas database. 9

Leave-one-out cross-comparison results: Jaccard overlaps

Supplementary Table C1. Jaccard overlap comparisons in HM atlas. 11
Supplementary Table C2. Jaccard overlap comparisons in DKT atlas. 13
Supplementary Table C3. Jaccard overlap comparisons in MGC2012 atlas. 14

Comparisons of group differences in region volumes for ADNI data

Supplementary Table D1.Group differences for the HM atlas database. 16
Supplementary Table D2. Group differences for the DKT40 atlas database. 18
Supplementary Table D3. Group differences for the MGC2012 atlas database. 20

Comparisons of group differences in region volumes for HS data

Supplementary Table E1.Group differences for the HM atlas database (left HS). 23
Supplementary Table E2. Group differences for the DKT40 atlas database (left HS). 25
Supplementary Table E3. Group differences for the MGC2012 atlas database (left HS)..................27
Supplementary Table E4. Group differences for the HM atlas database (right HS). 30
Supplementary Table E5. Group differences for the DKT40 atlas database (right HS). 32
Supplementary Table E6. Group differences for the MGC2012 atlas database (right HS). 34

FIGURES

Supplementary Figure S1. Boxplots of Jaccard overlaps for the HM atlas database. 37
Supplementary Figure S2. Boxplots of Jaccard overlaps for the DKT40 atlas database. 38
Supplementary Figure S3. Boxplots of Jaccard overlap for the MGC2012 atlas database. 39

Supplementary Methods: Masking to achieve compatibility between HM and the other atlases

We carried out brain extraction in five steps: 1) binarizing the manually generated labels to generate Mask A for later use, 2) generating a brain mask with FSL BET on each full image with -f and -g parameters optimized per subject to achieve visually satisfactory masking, 3) eroding the result of Step 2 in four iterations to generate Mask B, 4) combining Mask A and Mask B using Boolean OR, 5) multiplying the MR image with the mask resulting from Step 4.

Supplementary Table A1. Summary of atlas databases.

	HM	DKT40	MGC2012
**Scanner**	1.5T GE Signa Echospeed	n=19: 1.5T Siemens Vision<br>n=11: 3T Philips Achieva<br>n=10: 3T Phillips	1.5T Siemens Vision
**Sequence**	3D Cor IR-SPGR	n=19: 3D Sag MP-RAGE<br>n=11: 3D Sag MP-RAGE<br>n=10: 3D Unknown	3D Sag MP-RAGE
**Voxel Size (mm)**	0.9375×0.9375×1.5	n=19: 1×1×1.25<br>n=11: 1×1×1.2<br>n=10: 1×1×1	1×1×1.25
**Number of Regions**	95	62	138
**Atlas Voxel Size (mm)**	0.9375×0.9375×0.9375	n=29: 1×1×1<br>n=11: 1×1×1.2	1×1×1
**Number of Subjects**	30	40	30
**Age Range (mean ± SD)**	20–54 (31.0 ± 7.8)	19–61 (26.4 ± 7.3)	18–90 (34.3 ± 20.7)
**Gender (F/M)**	15/15	20/20	20/10
**Handedness (R/L)**	25/5	37/1[†]	30/0

Cor: coronal, Sag: sagittal, IR-SPGR: inversion recovery prepared fast spoiled gradient recall sequence, MP-RAGE: magnetization prepared rapid gradient-echo.
[†]2 unknown handedness

Supplementary Table A2. List of 95 cortical and sub-cortical labels for HM atlas.

Abbreviations: L = left, R = right, gyr. = gyrus, cort. = cortex, ant. = anterior, post. = posterior, sup. = superior, inf. = inferior, med. = medial, lat. = lateral, temp. = temporal.

No.	Region Name						
1	hippocampus R	24	cingulate gyrus, ant. L	48	lat. ventricle, temp. horn L	72	post. orbital gyr. L
2	hippocampus L	25	cingulate gyrus, ant. R	49	third ventricle	73	post. orbital gyr. R
3	amygdala R	26	cingulate gyrus, post. L	50	precentral gyr. L	74	substantia nigra L
4	amygdala L	27	cingulate gyrus, post. R	51	precentral gyr. R	75	substantia nigra R
5	ant. temp. lobe, med. R	28	middle frontal gyr. L	52	straight gyr. L	76	subgenual frontal cort. L
6	ant. temp. lobe, med. L	29	middle frontal gyr. R	53	straight gyr. R	77	subgenual frontal cort. R
7	ant. temp. lobe, lat. R	30	post. temp. lobe L	54	ant. orbital gyr. L	78	subcallosal area L
8	ant. temp. lobe, lat. L	31	post. temp. lobe R	55	ant. orbital gyr. R	79	subcallosal area R
9	parahippocampal gyr. R	32	angular gyr. L	56	inf. frontal gyr. L	80	pre-subgenual frontal cort. L
10	parahippocampal gyr. L	33	angular gyr. R	57	inf. frontal gyr. R	81	pre-subgenual frontal cort. R
11	sup. temp. gyr., post. R	34	caudate nucleus L	58	sup. frontal gyr. L	82	sup. temp. gyr., ant. L
12	sup. temp. gyr., post. L	35	caudate nucleus R	59	sup. frontal gyr. R	83	sup. temp. gyr., ant. R
13	middle & inf. temp. gyr. R	36	nucleus accumbens L	60	postcentral gyr. L	84	supramarginal gyr. L
14	middle & inf. temp. gyr. L	37	nucleus accumbens R	61	postcentral gyr. R	85	supramarginal gyr. R
15	fusiform gyr. R	38	putamen L	62	sup. parietal gyr. L	86	insula, ant. short gyr. L
16	fusiform gyr. L	39	putamen R	63	sup. parietal gyr. R	87	insula, ant. short gyr. R
17	cerebellum R	40	thalamus L	64	lingual gyr. L	88	insula, middle short gyr. L
18	cerebellum L	41	thalamus R	65	lingual gyr. R	89	insula, middle short gyr. R
19	brainstem	42	pallidum L	66	cuneus L	90	insula, post. short gyr. L
20	insula, post. long gyr. L	43	pallidum R	67	cuneus R	91	insula, post. short gyr. R
21	insula, post. long gyr. R	44	corpus callosum	68	med. orbital gyr. L	92	insula, ant. inf. cort. L
22	occipital lobe L	45	lat. ventricle, main R	69	med. orbital gyr. R	93	insula, ant. inf. cort. R
23	occipital lobe R	46	lat. ventricle, main L	70	lat. orbital gyr. L	94	insula, ant. long gyr. L
		47	lat. ventricle, temp. horn R	71	lat. orbital gyr. R	95	insula, ant. long gyr. R

Supplementary Table A3. List of 62 cortical labels for DKT40 atlas.

Abbreviations: L = left, R = right, gyr. = gyrus, cort. = cortex, ant. = anterior, post. = posterior, sup. = superior, inf. = inferior, med. = medial, lat. = lateral, temp. = temporal.

No.	Region Name	No.	Region Name
1	cingulate gyr., caudal ant. L	32	cingulate gyr., caudal ant. R
2	middle frontal gyr., caudal L	33	middle frontal gyr., caudal R
3	cuneus L	34	cuneus R
4	entorhinal cort. L	35	entorhinal cort. R
5	fusiform gyr. L	36	fusiform gyr. R
6	inf. parietal gyr. L	37	inf. parietal gyr. R
7	inf. temp. gyr. L	38	inf. temp. gyr. R
8	cingulate gyr., isthmus L	39	cingulate gyr., isthmus R
9	lat. occipital lobe L	40	lat. occipital lobe R
10	lat. orbitofrontal L	41	lat. orbitofrontal R
11	lingual gyr. L	42	lingual gyr. R
12	med. orbitofrontal L	43	med. orbitofrontal R
13	middle temp. gyr. L	44	middle temp. gyr. R
14	parahippocampal gyr. L	45	parahippocampal gyr. R
15	paracentral gyr. L	46	paracentral gyr. R
16	pars opercularis L	47	pars opercularis R
17	pars orbitalis L	48	pars orbitalis R
18	pars triangularis L	49	pars triangularis R
19	pericalcarine cort. L	50	pericalcarine cort. R
20	postcentral gyr. L	51	postcentral gyr. R
21	cingulate gyr., post. L	52	cingulate gyr., post. R
22	precentral gyr. L	53	precentral gyr. R
23	precuneus L	54	precuneus R
24	cingulate gyr., rostral ant. L	55	cingulate gyr., rostral ant. R
25	middle frontal gyr., rostral L	56	middle frontal gyr., rostral R
26	sup. frontal gyr. L	57	sup. frontal gyr. R
27	sup. parietal gyr. L	58	sup. parietal gyr. R
28	sup. temp. gyr. L	59	sup. temp. gyr. R
29	supramarginal gyr. L	60	supramarginal gyr. R
30	transverse temp. gyr. L	61	transverse temp. gyr. R
31	insula cort. L	62	insula cort. R

Supplementary Table A4. List of 132 cortical and sub-cortical labels for MGC2012 atlas.

Abbreviations: L = left, R = right, gyr. = gyrus, cort. = cortex, ant. = anterior, post. = posterior, sup. = superior, inf. = inferior, med. = medial, lat. = lateral, temp. = temporal.

No.	Region Name
1	third ventricle
2	fourth ventricle
3	accumbens area R
4	accumbens area L
5	amygdala R
6	amygdala L
7	brainstem
8	caudate R
9	caudate L
10	cerebellum exterior R
11	cerebellum exterior L
12	cerebellar white matter R
13	cerebellar white matter L
14	cerebral white matter R
15	cerebral white matter L
16	cerebrospinal fluid
17	hippocampus R
18	hippocampus L
19	inf. lateral ventricle R
20	inf. lateral ventricle L
21	lateral ventricle R
22	lateral ventricle L
23	pallidum R
24	pallidum L
25	putamen R
26	putamen L
27	thalamus (proper) R
28	thalamus (proper) L
29	ventral diencephalon R
30	ventral diencephalon L
31	optic chiasm
32	cerebellar vermal lobules I-V
33	cerebellar vermal lobules VI-VII
34	cerebellar vermal lobules VIII-X
35	cingulate gyrus, ant. R
36	cingulate gyrus, ant. L
37	ant. insula R
38	ant. insula L
39	ant. orbital gyr. R
40	ant. orbital gyr. L
41	angular gyrus R
42	angular gyrus L
43	calcarine cort. R
44	calcarine cort. L
45	central operculum R
46	central operculum L
47	cuneus R
48	cuneus L
49	entorhinal area R
50	entorhinal area L
51	frontal operculum R
52	frontal operculum L
53	frontal pole R
54	frontal pole L
55	fusiform gyr. R
56	fusiform gyr. L
57	gyrus rectus R
58	gyrus rectus L
59	inf. occipital gyr. R
60	inf. occipital gyr. L
61	inf. temp. gyr. R
62	inf. temp. gyr. L
63	lingual gyr. R
64	lingual gyr. L
65	lat. orbital gyr. R
66	lat. orbital gyr. L
67	cingulate gyrus, middle R
68	cingulate gyrus, middle L
69	med. frontal cort. R
70	med. frontal cort. L
71	middle frontal gyr. R
72	middle frontal gyr. L
73	middle occipital gyr. R
74	middle occipital gyr. L
75	med. orbital gyr. R
76	med. orbital gyr. L
77	postcentral gyr., med. R
78	postcentral gyr., med. L
79	precentral gyr., med. R
80	precentral gyr., med. L
81	sup. frontal gyr., med. R
82	sup. frontal gyr., med. L
83	middle temp. gyr. R
84	middle temp. gyr. L
85	occipital pole R
86	occipital pole L
87	occipital fusiform gyr. R
88	occipital fusiform gyr. L
89	inf. frontal gyr., opercular R
90	inf. frontal gyr., opercular L
91	inf. frontal gyr., orbital R
92	inf. frontal gyr., ortibal L
93	cingulate gyrus, post. R
94	cingulate gyrus, post. L
95	precuneus R
96	precuneus L
97	parahippocampal gyr. R
98	parahippocampal gyr. L
99	post. insula R
100	post. insula L
101	parietal operculum R
102	parietal operculum L
103	postcentral gyr. R
104	postcentral gyr. L
105	post. orbital gyr. R
106	post. orbital gyr. L
107	planum polare R
108	planum polare L
109	precentral gyr. R
110	precentral gyr. L
111	planum temporale R
112	planum temporale L
113	subcallosal area R
114	subcallosal area L
115	sup. frontal gyr. R
116	sup. frontal gyr. L
117	supplementary motor cort. R
118	supplementary motor cort. L
119	supramarginal gyr. R
120	supramarginal gyr. L
121	sup. occipital gyr. R
122	sup. occipital gyr. L
123	sup. parietal lobule R
124	sup. parietal lobule L
125	sup. temp. gyr. R
126	sup. temp. gyr. L
127	temporal pole R
128	temporal pole L
129	inf. frontal gyr., triangular R
130	inf. frontal gyr., triangular L
131	transverse temp. gyr. R
132	transverse temp. gyr. L

Supplementary Table B1. Region properties in HM atlas database.

Region volumes (mm^3), coefficient of variation (CV) and surface-to-volume ratio (SVR) for all subjects in the HM atlas database. Volumes are expressed as a fraction of ICV multiplied by 10^4. Abbreviations: gyr. = gyrus, cort. = cortex, ant. = anterior, post. = posterior, sup. = superior, inf. = inferior, med. = medial, lat. = lateral, temp. = temporal. np: non-paired regions.

	Right Hemisphere			Left Hemisphere		
**Region**	**Volume (mean ± SD)**	**CV**	**SVR (mean ± SD)**	**Volume (mean ± SD)**	**CV**	**SVR (mean ± SD)**
Temporal lobe						
hippocampus	13.6 ± 1.79	0.132	0.81 ± 0.11	12.7 ± 1.62	0.128	0.84 ± 0.09
amygdala	9.35 ± 1.39	0.149	0.69 ± 0.05	9.98 ± 1.91	0.191	0.67 ± 0.08
ant. temp. lobe, med.	27.6 ± 5.95	0.216	1.11 ± 0.21	26.7 ± 4.54	0.170	1.03 ± 0.17
ant. temp. lobe, lat.	14.3 ± 3.88	0.271	1.27 ± 0.22	13.7 ± 3.75	0.274	1.17 ± 0.17
parahippocampal gyr.	18.5 ± 2.40	0.130	1.25 ± 0.11	19.5 ± 2.13	0.109	1.27 ± 0.11
sup. temp. gyr., post.	42.9 ± 5.29	0.123	1.24 ± 0.09	42.9 ± 5.76	0.134	1.03 ± 0.08
middle & inf. temp. gyr.	55.4 ± 6.58	0.119	1.17 ± 0.13	53.1 ± 8.23	0.155	1.13 ± 0.12
fusiform gyr.	16.1 ± 2.84	0.176	1.37 ± 0.16	17.0 ± 3.47	0.205	1.24 ± 0.14
post. temp. lobe	146 ± 16.9	0.116	1.03 ± 0.08	141 ± 19.6	0.139	1.07 ± 0.09
sup. temp. gyr., ant.	19.3 ± 3.81	0.198	1.21 ± 0.17	20.3 ± 4.60	0.227	1.01 ± 0.14
Insula and cingulate gyri						
insula, post. long gyr.	7.74 ± 1.49	0.192	1.26 ± 0.15	7.66 ± 1.34	0.175	1.27 ± 0.11
insula, ant. short gyr.	5.96 ± 1.39	0.233	1.17 ± 0.15	6.58 ± 1.05	0.160	1.19 ± 0.13
insula, middle short gyr.	3.60 ± 1.26	0.350	1.30 ± 0.19	3.69 ± 0.83	0.224	1.36 ± 0.12
insula, post. short gyr.	5.85 ± 1.28	0.218	1.16 ± 0.13	5.70 ± 1.37	0.239	1.18 ± 0.10
insula, ant. inf. cort.	8.07 ± 2.45	0.304	1.28 ± 0.22	8.50 ± 2.51	0.296	1.18 ± 0.18
insula, ant. long gyr.	8.34 ± 1.26	0.151	1.17 ± 0.10	8.43 ± 1.45	0.172	1.16 ± 0.12
cingulate gyrus, ant.	27.6 ± 6.96	0.252	1.06 ± 0.13	28.6 ± 7.06	0.247	1.09 ± 0.14
cingulate gyrus, post.	28.7 ± 4.82	0.168	1.04 ± 0.09	28.0 ± 3.61	0.129	1.12 ± 0.11
Frontal lobe						
middle frontal gyr.	122 ± 18.4	0.151	1.16 ± 0.08	128 ± 16.7	0.131	1.03 ± 0.07
precentral gyr.	84.1 ± 11.2	0.133	1.25 ± 0.09	87.2 ± 8.49	0.097	1.17 ± 0.09
ant. orbital gyr.	21.0 ± 3.82	0.182	1.27 ± 0.13	20.4 ± 3.73	0.183	1.23 ± 0.13
inf. frontal gyr.	56.3 ± 11.3	0.200	1.13 ± 0.11	61.0 ± 11.2	0.183	1.09 ± 0.09
sup. frontal gyr.	161 ± 22.1	0.138	1.02 ± 0.08	160 ± 22.6	0.141	1.03 ± 0.08
med. orbital gyr.	18.5 ± 3.71	0.201	1.56 ± 0.17	19.5 ± 4.25	0.218	1.50 ± 0.16
lat. orbital gyr.	15.3 ± 5.56	0.364	1.29 ± 0.15	14.3 ± 4.89	0.342	1.36 ± 0.15
post. orbital gyr.	17.5 ± 3.16	0.181	1.22 ± 0.13	17.9 ± 3.75	0.209	1.19 ± 0.15
subgenual frontal cort.	3.14 ± 1.15	0.366	1.48 ± 0.26	3.49 ± 1.10	0.314	1.45 ± 0.27
pre-subgenual frontal cort.	3.16 ± 1.49	0.471	1.42 ± 0.21	4.20 ± 1.66	0.396	1.29 ± 0.19
Occipital lobe						
lingual gyr.	43.8 ± 7.00	0.160	1.36 ± 0.10	43.4 ± 7.41	0.171	1.33 ± 0.10
cuneus	32.6 ± 4.99	0.153	1.43 ± 0.12	33.7 ± 6.86	0.203	1.43 ± 0.12
occipital lobe	134 ± 27.5	0.205	1.16 ± 0.08	133 ± 21.7	0.163	1.17 ± 0.06
Parietal lobe						
straight gyr.	11.9 ± 2.71	0.227	1.64 ± 0.15	10.7 ± 2.22	0.208	1.57 ± 0.15
postcentral gyr.	70.4 ± 8.09	0.115	1.42 ± 0.09	76.3 ± 7.17	0.094	1.31 ± 0.09
sup. parietal gyr.	123 ± 13.7	0.111	1.16 ± 0.07	121 ± 15.9	0.132	1.10 ± 0.08
supramarginal gyr.	67.4 ± 13.6	0.201	1.10 ± 0.08	74.5 ± 11.8	0.158	1.03 ± 0.07
angular gyr.	55.5 ± 11.6	0.210	1.03 ± 0.07	52.1 ± 14.9	0.285	1.06 ± 0.08
Central structures						
caudate nucleus	27.1 ± 3.15	0.116	0.66 ± 0.03	27.3 ± 3.28	0.120	0.56 ± 0.03
nucleus accumbens	2.08 ± 0.39	0.188	1.18 ± 0.13	2.41 ± 0.52	0.217	1.11 ± 0.09
putamen	29.8 ± 2.58	0.086	0.56 ± 0.03	31.2 ± 2.70	0.087	0.60 ± 0.03

thalamus	45.8 ± 4.35	0.095	0.42 ± 0.03	47.1 ± 4.62	0.098	0.43 ± 0.03
pallidum	8.72 ± 1.19	0.137	0.78 ± 0.07	8.77 ± 1.17	0.133	0.81 ± 0.04
corpus callosum (np)	135 ± 18.7	0.139	0.56 ± 0.05			
substantia nigra	2.44 ± 0.45	0.184	1.39 ± 0.13	2.51 ± 0.41	0.163	1.27 ± 0.11
subcallosal area	2.09 ± 0.71	0.340	1.66 ± 0.27	2.29 ± 0.80	0.348	1.60 ± 0.28
Posterior fossa & ventricles						
cerebellum	460 ± 41.8	0.091	0.20 ± 0.01	461 ± 38.4	0.083	0.20 ± 0.01
brainstem (np)	160 ± 9.81	0.061	0.34 ± 0.02			
lat. ventricle, main	51.1 ± 22.3	0.436	0.75 ± 0.17	56.2 ± 20.9	0.372	0.69 ± 0.14
lat. ventricle, temp. horn	4.60 ± 0.87	0.189	1.57 ± 0.19	3.86 ± 0.80	0.207	1.80 ± 0.24
third ventricle (np)	6.68 ± 1.96	0.294	1.11 ± 0.20			

Supplementary Table B2. Region properties in DKT atlas database.

Region volumes (mm^3), coefficient of variation (CV) and surface-to-volume ratio (SVR) for all subjects in the DKT40 atlas database. Volumes are expressed as a fraction of ICV multiplied by 10^4. Abbreviations as in Supp. Table B1.

Region	Right Hemisphere			Left Hemisphere		
	Volume (mean ± SD)	CV	SVR (mean ± SD)	Volume (mean ± SD)	CV	SVR (mean ± SD)
Temporal lobe						
entorhinal cort.	12.0 ± 2.33	0.193	0.83 ± 0.22	12.4 ± 2.35	0.190	0.84 ± 0.22
parahippocampal gyr.	14.0 ± 2.76	0.197	0.96 ± 0.27	15.1 ± 2.80	0.185	0.93 ± 0.26
sup. temp. gyr.	106 ± 15.4	0.144	0.82 ± 0.20	115 ± 12.6	0.110	0.73 ± 0.19
middle temp. gyr.	94.2 ± 12.3	0.131	0.82 ± 0.19	95.0 ± 15.3	0.161	0.82 ± 0.20
inf. temp. gyr.	79.7 ± 9.70	0.122	0.80 ± 0.21	80.3 ± 10.7	0.133	0.83 ± 0.21
fusiform gyr.	56.5 ± 10.2	0.181	0.92 ± 0.27	60.9 ± 11.8	0.194	0.89 ± 0.26
transverse temp. gyr.	7.00 ± 1.30	0.186	1.16 ± 0.29	8.52 ± 2.04	0.239	1.03 ± 0.27
Insula and cingulate gyri						
insula cort.	40.9 ± 4.06	0.099	0.71 ± 0.25	40.7 ± 4.29	0.105	0.75 ± 0.26
cingulate gyr., rostral ant.	20.0 ± 8.06	0.404	0.94 ± 0.27	25.2 ± 7.37	0.292	0.94 ± 0.27
cingulate gyr., caudal ant.	20.3 ± 6.75	0.333	0.83 ± 0.26	22.8 ± 7.34	0.322	0.89 ± 0.27
cingulate gyr., post.	24.6 ± 4.61	0.187	0.93 ± 0.26	26.1 ± 4.79	0.183	0.99 ± 0.27
cingulate gyr., isthmus	16.4 ± 4.14	0.252	1.03 ± 0.28	16.2 ± 3.16	0.195	1.06 ± 0.29
Frontal lobe						
middle frontal gyr., rostral	78.9 ± 13.0	0.164	0.96 ± 0.22	79.4 ± 12.4	0.157	0.92 ± 0.208
middle frontal gyr., caudal	42.9 ± 7.06	0.165	0.93 ± 0.22	47.6 ± 7.79	0.164	0.85 ± 0.20
sup. frontal gyr.	176 ± 23.4	0.133	0.80 ± 0.22	166 ± 22.1	0.133	0.82 ± 0.22
pars opercularis	33.0 ± 8.76	0.265	0.93 ± 0.20	31.9 ± 10.1	0.317	0.91 ± 0.20
pars triangularis	29.7 ± 7.40	0.249	0.93 ± 0.21	31.0 ± 9.71	0.313	0.96 ± 0.19
pars orbitalis	16.6 ± 5.59	0.336	0.94 ± 0.25	15.8 ± 5.27	0.333	1.00 ± 0.25
lat. orbitofrontal	57.7 ± 6.76	0.117	0.88 ± 0.23	55.7 ± 8.92	0.160	0.89 ± 0.26
med. orbitofrontal	26.6 ± 4.07	0.153	1.04 ± 0.33	28.3 ± 6.02	0.213	1.00 ± 0.33
precentral gyr.	88.4 ± 12.0	0.136	0.89 ± 0.21	90.0 ± 11.2	0.125	0.84 ± 0.19
paracentral gyr.	28.8 ± 4.28	0.148	0.91 ± 0.28	27.7 ± 5.22	0.189	0.94 ± 0.28
Occipital lobe						
lingual gyr.	48.4 ± 6.83	0.141	1.06 ± 0.28	45.0 ± 10.3	0.228	1.10 ± 0.31
cuneus	28.5 ± 5.27	0.185	1.16 ± 0.33	29.3 ± 4.97	0.170	1.17 ± 0.36
lat. occipital lobe	81.4 ± 9.56	0.117	0.99 ± 0.22	78.2 ± 9.71	0.124	0.99 ± 0.23
pericalcarine cort.	15.3 ± 2.69	0.175	1.33 ± 0.31	13.8 ± 2.72	0.197	1.33 ± 0.33
Parietal lobe						
postcentral gyr.	66.0 ± 10.9	0.165	1.13 ± 0.26	71.0 ± 9.64	0.136	1.02 ± 0.24
sup. parietal gyr.	77.4 ± 9.80	0.127	1.00 ± 0.26	72.6 ± 12.0	0.165	0.94 ± 0.26
supramarginal gyr.	71.0 ± 14.1	0.199	0.90 ± 0.19	74.1 ± 13.5	0.182	0.85 ± 0.18
inf. parietal gyr.	92.3 ± 18.4	0.200	0.86 ± 0.22	81.5 ± 16.2	0.199	0.88 ± 0.22
precuneus	68.9 ± 10.2	0.149	0.94 ± 0.25	65.3 ± 8.32	0.127	0.92 ± 0.26

Supplementary Table B3. Region properties in MGC2012 atlas database.

Region volumes (mm^3), coefficient of variation (CV) and surface-to-volume ratio (SVR) for all subjects in the MGC2012 atlas database. Volumes are expressed as a fraction of ICV multiplied by 10^4. Abbreviations as in Supp. Table B1.

Region	Right Hemisphere			Left Hemisphere		
	Volume (mean ± SD)	CV	SVR (mean ± SD)	Volume (mean ± SD)	CV	SVR (mean ± SD)
Temporal lobe						
hippocampus	25.1 ± 2.63	0.105	0.56 ± 0.02	23.8 ± 2.60	0.109	0.62 ± 0.02
amygdala	6.74 ± 1.08	0.161	0.70 ± 0.05	6.62 ± 0.97	0.146	0.75 ± 0.06
entorhinal area	13.0 ± 3.45	0.265	0.78 ± 0.07	12.9 ± 3.38	0.262	0.73 ± 0.08
parahippocampal gyr.	21.0 ± 3.93	0.187	0.86 ± 0.06	22.5 ± 2.76	0.123	0.88 ± 0.04
sup. temp. gyr.	56.3 ± 9.04	0.160	0.77 ± 0.06	55.2 ± 12.5	0.226	0.70 ± 0.05
middle temp. gyr.	107 ± 12.4	0.116	0.64 ± 0.04	104 ± 16.6	0.159	0.64 ± 0.05
inf. temp. gyr.	84.8 ± 11.3	0.134	0.62 ± 0.04	84.3 ± 14.3	0.170	0.70 ± 0.06
fusiform gyr.	55.1 ± 11.0	0.199	0.72 ± 0.05	55.4 ± 12.0	0.217	0.73 ± 0.07
transverse temp. gyr.	11.5 ± 3.77	0.329	1.01 ± 0.09	12.3 ± 3.82	0.309	0.90 ± 0.10
temporal pole	59.2 ± 12.0	0.202	0.60 ± 0.04	58.6 ± 11.1	0.190	0.56 ± 0.04
planum temporale	14.7 ± 4.61	0.313	1.05 ± 0.09	15.8 ± 4.20	0.265	0.87 ± 0.08
planum polare	15.0 ± 4.62	0.308	1.06 ± 0.09	16.3 ± 4.16	0.255	0.82 ± 0.05
Insula and cingulate gyri						
ant. insula	30.2 ± 2.34	0.077	0.64 ± 0.03	31.1 ± 4.10	0.132	0.72 ± 0.04
post. insula	16.8 ± 2.20	0.131	0.77 ± 0.06	16.4 ± 2.76	0.169	0.75 ± 0.06
cingulate gyrus, ant.	31.3 ± 8.59	0.274	0.68 ± 0.04	36.4 ± 10.7	0.295	0.67 ± 0.05
cingulate gyrus, middle	35.9 ± 4.79	0.133	0.72 ± 0.05	36.8 ± 7.69	0.209	0.73 ± 0.07
cingulate gyrus, post.	30.7 ± 7.56	0.246	0.80 ± 0.08	33.6 ± 7.26	0.216	0.82 ± 0.06
Frontal lobe						
middle frontal gyr.	141 ± 18.9	0.134	0.68 ± 0.06	144 ± 16.9	0.117	0.63 ± 0.05
precentral gyr.	96.4 ± 10.2	0.106	0.76 ± 0.06	97.2 ± 14.1	0.145	0.71 ± 0.06
precentral gyr., med.	19.9 ± 5.20	0.262	0.81 ± 0.08	20.5 ± 5.18	0.253	0.86 ± 0.08
sup. frontal gyr.	111 ± 21.4	0.194	0.70 ± 0.06	109 ± 15.3	0.140	0.73 ± 0.06
sup. frontal gyr., med.	59.0 ± 10.9	0.185	0.69 ± 0.05	52.9 ± 13.3	0.252	0.74 ± 0.07
med. frontal cort.	14.2 ± 4.34	0.305	1.01 ± 0.11	14.6 ± 3.62	0.247	0.89 ± 0.07
inf. frontal gyr., opercular	26.3 ± 8.20	0.311	0.84 ± 0.09	25.1 ± 7.43	0.296	0.83 ± 0.08
inf. frontal gyr., orbital	12.3 ± 4.57	0.373	0.92 ± 0.10	11.8 ± 3.92	0.332	1.05 ± 0.11
inf. frontal gyr., triangular	27.5 ± 8.35	0.303	0.80 ± 0.08	29.4 ± 8.33	0.283	0.84 ± 0.07
frontal operculum	14.8 ± 4.16	0.281	0.82 ± 0.08	14.9 ± 4.12	0.277	1.00 ± 0.09
frontal pole	27.8 ± 11.7	0.422	0.80 ± 0.14	26.0 ± 13.3	0.509	0.83 ± 0.10
central operculum	29.6 ± 7.92	0.268	0.73 ± 0.05	29.8 ± 5.94	0.199	0.79 ± 0.06
ant. orbital gyr.	14.3 ± 4.15	0.291	0.87 ± 0.08	12.0 ± 3.55	0.296	0.89 ± 0.08
post. orbital gyr.	18.0 ± 4.31	0.239	0.86 ± 0.08	19.6 ± 4.99	0.254	0.85 ± 0.08
med. orbital gyr.	31.3 ± 4.83	0.154	0.84 ± 0.08	32.1 ± 6.37	0.199	0.74 ± 0.06
lat. orbital gyr.	17.8 ± 5.04	0.283	0.88 ± 0.10	18.5 ± 5.95	0.322	0.96 ± 0.09
supplementary motor cort.	39.8 ± 8.15	0.205	0.70 ± 0.05	40.8 ± 7.37	0.181	0.76 ± 0.07
Occipital lobe						
lingual gyr.	61.5 ± 15.3	0.249	0.79 ± 0.08	57.1 ± 14.3	0.251	0.80 ± 0.10
cuneus	37.8 ± 7.86	0.208	0.86 ± 0.08	35.2 ± 8.75	0.249	0.85 ± 0.09
sup. occipital gyr.	33.6 ± 11.0	0.328	0.84 ± 0.09	27.5 ± 8.63	0.314	0.87 ± 0.09
middle occipital gyr.	41.6 ± 10.2	0.246	0.71 ± 0.06	47.2 ± 12.4	0.263	0.68 ± 0.07
inf. occipital gyr.	50.8 ± 12.4	0.245	0.76 ± 0.08	47.8 ± 12.4	0.260	0.73 ± 0.09
occipital pole	22.5 ± 10.2	0.454	0.85 ± 0.16	26.4 ± 12.1	0.458	0.76 ± 0.09
occipital fusiform gyr.	31.9 ± 9.06	0.284	0.84 ± 0.08	32.4 ± 10.6	0.327	0.83 ± 0.10
calcarine cort.	25.6 ± 6.02	0.235	0.74 ± 0.07	25.6 ± 4.90	0.191	0.74 ± 0.08
Parietal lobe						

gyrus rectus	16.7 ± 3.98	0.239	0.95 ± 0.11	18.1 ± 3.77	0.209	0.80 ± 0.09
postcentral gyr.	78.6 ± 12.0	0.153	0.88 ± 0.09	88.9 ± 17.8	0.200	0.79 ± 0.08
postcentral gyr., med.	7.90 ± 3.72	0.470	0.98 ± 0.11	8.56 ± 4.81	0.562	1.05 ± 0.15
sup. parietal lobule	78.2 ± 10.6	0.135	0.75 ± 0.07	79.2 ± 12.3	0.155	0.70 ± 0.06
supramarginal gyr.	63.5 ± 16.1	0.253	0.73 ± 0.06	69.0 ± 16.7	0.243	0.70 ± 0.06
precuneus	78.9 ± 10.5	0.133	0.69 ± 0.06	80.0 ± 10.8	0.135	0.67 ± 0.05
parietal operculum	17.1 ± 4.90	0.287	0.86 ± 0.08	18.6 ± 4.75	0.256	0.81 ± 0.10
angular gyrus	79.7 ± 25.1	0.315	0.63 ± 0.07	70.0 ± 20.1	0.288	0.66 ± 0.06
Central structures						
caudate	22.5 ± 2.65	0.118	0.66 ± 0.06	21.9 ± 2.98	0.137	0.54 ± 0.05
accumbens area	3.18 ± 0.90	0.284	1.08 ± 0.16	3.54 ± 0.82	0.232	1.11 ± 0.10
putamen	30.3 ± 2.90	0.095	0.50 ± 0.04	31.5 ± 3.10	0.099	0.53 ± 0.04
thalamus (proper)	52.5 ± 4.68	0.089	0.36 ± 0.02	54.9 ± 5.05	0.092	0.38 ± 0.02
pallidum	10.4 ± 1.46	0.140	0.63 ± 0.09	10.3 ± 1.10	0.106	0.70 ± 0.04
ventral diencephalon	32.0 ± 2.39	0.075	0.51 ± 0.03	33.7 ± 2.53	0.075	0.51 ± 0.03
optic chiasm (np)	0.57 ± 0.21	0.362	1.85 ± 0.38			
subcallosal area	9.00 ± 3.54	0.393	0.89 ± 0.12	8.98 ± 4.09	0.455	0.93 ± 0.14
Posterior fossa, ventricles & white matter						
cerebellum exterior	350 ± 28.2	0.080	0.33 ± 0.02	350 ± 28.5	0.082	0.34 ± 0.02
cerebellar vermal lobules I-V (np)	33.4 ± 7.74	0.232	0.54 ± 0.09			
cerebellar vermal lobules VI-VII (np)	16.0 ± 3.90	0.243	0.69 ± 0.07			
cerebellar vermal lobules VIII-X (np)	19.8 ± 3.34	0.169	0.59 ± 0.06			
brainstem	123 ± 11.2	0.091	0.32 ± 0.01			
lateral ventricle	61.8 ± 44.7	0.724	0.62 ± 0.19	74.4 ± 54.3	0.731	0.58 ± 0.18
inf. lateral ventricle	3.10 ± 1.43	0.462	1.45 ± 0.29	2.63 ± 1.41	0.536	1.73 ± 0.41
third ventricle (np)	5.85 ± 3.98	0.680	1.10 ± 0.30			
fourth ventricle (np)	12.3 ± 3.67	0.297	0.92 ± 0.12			
cerebrospinal fluid (np)	7.09 ± 1.86	0.262	0.92 ± 0.08			
cerebral white matter	1450 ± 121	0.084	0.44 ± 0.03	1430 ± 119	0.083	0.44 ± 0.03
cerebellar white matter	95.8 ± 13.8	0.144	0.52 ± 0.04	98.0 ± 10.9	0.112	0.54 ± 0.04

Supplementary Table C1. Jaccard overlap comparisons in HM atlas.

Leave-one-out cross-comparison results for MAPER and FreeSurfer segmentation of the Hammers_mith atlas. The mean JC (across 30 subjects) for each region is shown, with significant differences in JC between MAPER and FreeSurfer (from Welch's two-tailed paired t-test, after adjustment for 93 multiple comparisons) shown in bold. Abbreviations as in Supp. Table B1.

**Region**	**Right hemisphere**		**Left hemisphere**	
	**MAPER**	**FreeSurfer**	**MAPER**	**FreeSurfer**
Temporal lobe				
hippocampus	**0.728**	0.664	**0.712**	0.643
amygdala	**0.667**	0.628	0.662	0.626
ant. temp. lobe, med.	**0.793**	0.707	**0.797**	0.693
ant. temp. lobe, lat.	**0.682**	0.520	**0.677**	0.452
parahippocampal gyr.	0.722	0.758	0.705	**0.767**
sup. temp. gyr., post.	0.838	0.802	**0.845**	0.803
middle & inf. temp. gyr.	0.826	0.783	**0.808**	0.731
fusiform gyr.	**0.676**	0.580	**0.648**	0.535
post. temp. lobe	**0.762**	0.709	**0.778**	0.715
sup. temp. gyr., ant.	0.762	0.726	0.775	0.713
Insula and cingulate gyri				
insula, post. long gyr.	**0.755**	0.709	**0.748**	0.695
insula, ant. short gyr.	**0.730**	0.669	**0.753**	0.669
insula, middle short gyr.	**0.625**	0.531	**0.682**	0.545
insula, post. short gyr.	**0.753**	0.628	**0.737**	0.598
insula, ant. inf. cort.	**0.690**	0.637	**0.697**	0.640
insula, ant. long gyr.	**0.760**	0.627	**0.744**	0.634
cingulate gyrus, ant.	0.778	0.780	0.721	0.721
cingulate gyrus, post.	0.750	0.771	0.787	0.799
Frontal lobe				
middle frontal gyr.	0.793	0.796	0.787	0.784
precentral gyr.	0.775	0.766	0.770	0.766
ant. orbital gyr.	0.722	0.702	0.675	0.645
inf. frontal gyr.	0.780	0.759	0.778	0.760
sup. frontal gyr.	0.830	0.837	0.821	0.822
med. orbital gyr.	0.633	0.604	**0.628**	0.563
lat. orbital gyr.	0.490	0.465	0.438	0.440
post. orbital gyr.	0.708	0.678	0.680	0.645
subgenual frontal cort.	**0.666**	0.573	**0.688**	0.559
pre-subgenual frontal cort.	0.552	0.497	0.598	0.514
Occipital lobe				
lingual gyr.	0.733	**0.778**	0.759	0.802
cuneus	0.750	0.773	0.747	0.753
occipital lobe	0.725	0.729	0.746	0.738
Parietal lobe				
straight gyr.	0.719	0.692	0.704	0.650
postcentral gyr.	0.765	0.748	0.776	0.781
sup. parietal gyr.	0.820	0.826	0.817	0.837
supramarginal gyr.	0.718	0.690	0.779	0.753
angular gyr.	0.595	0.558	0.645	0.595
Central structures				
caudate nucleus	**0.813**	0.743	**0.809**	0.740
nucleus accumbens	0.521	0.490	0.550	0.502

putamen	**0.815**	0.752	**0.809**	0.753
thalamus	0.803	0.783	**0.806**	0.776
pallidum	0.668	0.617	**0.667**	0.623
corpus callosum (np)	**0.770**	0.718		
substantia nigra	**0.588**	0.526	**0.586**	0.501
Posterior fossa & ventricles				
cerebellum	**0.930**	0.892	**0.933**	0.892
brainstem (np)	**0.883**	0.840		
lat. ventricle, main	**0.818**	0.734	**0.835**	0.752
lat. ventricle, temp. horn	**0.529**	0.434	**0.493**	0.396
third ventricle (np)	**0.711**	0.641		

Supplementary Table C2. Jaccard overlap comparisons in DKT atlas.

Leave-one-out cross-comparison results for MAPER and FreeSurfer segmentation of the Desikan-Killiany-Tourville 40 atlas. The mean JC (across 30 subjects) for each region is shown, with significant differences in JC between MAPER and FreeSurfer (from Welch's two-tailed paired t-test, after adjustment for 62 multiple comparisons) shown in bold. Abbreviations as in Supp. Table B1.

**Region**	**Right hemisphere**		**Left hemisphere**	
	**MAPER**	**FreeSurfer**	**MAPER**	**FreeSurfer**
Temporal lobe				
entorhinal cort.	0.534	**0.605**	0.546	**0.644**
parahippocampal gyr.	0.631	**0.750**	0.651	**0.749**
sup. temp. gyr.	0.706	**0.792**	0.703	**0.802**
middle temp. gyr.	0.657	**0.776**	0.637	**0.749**
inf. temp. gyr.	0.598	**0.736**	0.592	**0.713**
fusiform gyr.	0.615	**0.705**	0.605	**0.693**
transverse temp. gyr.	0.635	**0.722**	0.612	**0.704**
Insula and cingulate gyri				
insula cort.	0.750	**0.821**	0.758	**0.812**
cingulate gyr., rostral ant.	0.567	0.610	0.604	0.621
cingulate gyr., caudal ant.	0.621	0.684	0.593	0.622
cingulate gyr., post.	0.644	**0.737**	0.626	**0.714**
cingulate gyr., isthmus	0.633	**0.737**	0.624	**0.731**
Frontal lobe				
middle frontal gyr., rostral	0.605	**0.738**	0.615	**0.736**
middle frontal gyr., caudal	0.569	**0.787**	0.590	**0.799**
sup. frontal gyr.	0.667	**0.769**	0.660	**0.757**
pars opercularis	0.632	0.683	0.590	**0.661**
pars triangularis	0.580	**0.643**	0.533	**0.574**
pars orbitalis	0.513	0.553	0.496	**0.545**
lat. orbitofrontal	0.671	**0.758**	0.655	**0.752**
med. orbitofrontal	0.583	**0.657**	0.580	**0.660**
precentral gyr.	0.646	**0.781**	0.643	**0.788**
paracentral gyr.	0.622	**0.764**	0.603	**0.736**
Occipital lobe				
lingual gyr.	0.634	**0.776**	0.605	**0.752**
cuneus	0.521	**0.644**	0.531	**0.670**
lat. occipital lobe	0.551	**0.700**	0.533	**0.706**
pericalcarine cort.	0.518	**0.705**	0.487	**0.705**
Parietal lobe				
postcentral gyr.	0.599	**0.725**	0.598	**0.743**
sup. parietal gyr.	0.572	**0.733**	0.574	**0.755**
supramarginal gyr.	0.605	**0.679**	0.623	**0.733**
inf. parietal gyr.	0.595	**0.709**	0.590	**0.732**
precuneus	0.669	**0.771**	0.670	**0.782**

Supplementary Table C3. Jaccard overlap comparisons in MGC2012 atlas.

Leave-one-out cross-comparison results for MAPER and FreeSurfer segmentation of the MICCAI 2012 Grand Challenge atlas. The mean JC (across 30 subjects) for each region is shown, with significant differences in JC between MAPER and FreeSurfer (from Welch's two-tailed paired t-test, after adjustment for 132 multiple comparisons) shown in bold. Abbreviations as in Supp. Table B1.

Region	Right hemisphere		Left hemisphere	
	MAPER	FreeSurfer	MAPER	FreeSurfer
Temporal lobe				
hippocampus	**0.747**	0.704	**0.746**	0.700
amygdala	**0.666**	0.599	**0.666**	0.604
entorhinal area	**0.544**	0.297	**0.544**	0.338
parahippocampal gyr.	**0.583**	0.459	**0.597**	0.487
sup. temp. gyr.	**0.680**	0.544	**0.618**	0.503
middle temp. gyr.	**0.675**	0.575	**0.649**	0.538
inf. temp. gyr.	**0.629**	0.547	0.578	0.537
fusiform gyr.	**0.636**	0.504	**0.610**	0.511
transverse temp. gyr.	**0.584**	0.456	**0.589**	0.472
temporal pole	**0.663**	0.580	**0.680**	0.599
planum temporale	**0.543**	0.426	**0.561**	0.441
planum polare	**0.588**	0.460	**0.589**	0.495
Insula and cingulate gyri				
ant. insula	**0.748**	0.632	**0.755**	0.627
post. insula	**0.707**	0.577	**0.694**	0.550
cingulate gyrus, ant.	**0.687**	0.502	**0.652**	0.459
cingulate gyrus, middle	**0.710**	0.539	**0.681**	0.509
cingulate gyrus, post.	**0.609**	0.494	**0.638**	0.499
Frontal lobe				
middle frontal gyr.	**0.699**	0.556	**0.724**	0.577
precentral gyr.	**0.717**	0.588	**0.713**	0.591
precentral gyr., med.	**0.548**	0.445	**0.584**	0.470
sup. frontal gyr.	**0.668**	0.578	**0.688**	0.588
sup. frontal gyr., med.	**0.656**	0.520	**0.636**	0.516
med. frontal cort.	**0.381**	0.283	**0.514**	0.348
inf. frontal gyr., opercular	**0.589**	0.451	**0.577**	0.448
inf. frontal gyr., orbital	0.402	0.362	**0.423**	0.348
inf. frontal gyr., triangular	**0.541**	0.437	**0.532**	0.441
frontal operculum	**0.547**	0.366	**0.549**	0.372
frontal pole	**0.527**	0.401	**0.489**	0.376
central operculum	**0.649**	0.509	**0.629**	0.495
ant. orbital gyr.	**0.454**	0.362	**0.431**	0.299
post. orbital gyr.	**0.600**	0.526	**0.619**	0.511
med. orbital gyr.	**0.637**	0.523	**0.620**	0.439
lat. orbital gyr.	0.419	0.364	0.443	0.376
supplementary motor cort.	**0.620**	0.522	**0.623**	0.518
Occipital lobe				
lingual gyr.	**0.601**	0.510	**0.610**	0.494
cuneus	**0.565**	0.427	**0.585**	0.415
sup. occipital gyr.	**0.509**	0.397	**0.482**	0.382
middle occipital gyr.	**0.538**	0.417	**0.516**	0.410
inf. occipital gyr.	**0.511**	0.451	**0.517**	0.424
occipital pole	**0.489**	0.325	**0.489**	0.334

occipital fusiform gyr.	**0.411**	0.343	**0.417**	0.349
calcarine cort.	**0.616**	0.430	**0.615**	0.424
Parietal lobe				
gyrus rectus	**0.522**	0.405	**0.563**	0.417
postcentral gyr.	**0.674**	0.500	**0.651**	0.491
postcentral gyr., med.	0.369	0.314	0.392	0.303
sup. parietal lobule	**0.618**	0.508	**0.637**	0.522
supramarginal gyr.	**0.573**	0.473	**0.560**	0.473
precuneus	**0.668**	0.537	**0.675**	0.516
parietal operculum	**0.557**	0.421	**0.552**	0.426
angular gyrus	**0.559**	0.459	**0.540**	0.455
Central structures				
caudate	**0.795**	0.745	**0.794**	0.729
accumbens area	**0.638**	0.550	**0.626**	0.554
putamen	**0.839**	0.789	**0.834**	0.788
thalamus (proper)	**0.845**	0.799	**0.851**	0.796
pallidum	**0.758**	0.711	**0.740**	0.685
ventral diencephalon	**0.795**	0.725	**0.794**	0.723
optic chiasm (np)	0.289	0.317		
subcallosal area	**0.510**	0.420	**0.475**	0.313
Posterior fossa, ventricles & white matter				
cerebellum exterior	0.847	0.833	0.843	0.822
cerebellar vermal lobules I-V (np)	**0.717**	0.671		
cerebellar vermal lobules VI-VII (np)	**0.642**	0.569		
cerebellar vermal lobules VIII-X (np)	**0.771**	0.682		
brainstem	**0.871**	0.840		
lateral ventricle	**0.855**	0.783	**0.860**	0.800
inf. lateral ventricle	0.420	0.431	0.379	0.386
third ventricle (np)	**0.736**	0.630		
fourth ventricle (np)	**0.764**	0.688		
cerebrospinal fluid (np)	**0.664**	0.580		
cerebral white matter	**0.887**	0.869	0.882	0.867
cerebellar white matter	0.790	0.792	0.786	0.788

Supplementary Table D1. Group differences for the HM atlas database.

Negative percentage difference values indicate smaller volumes in AD than HC. Significant differences in structure volumes between HC and AD groups (from the ANCOVA, after adjustment for 93 multiple comparisons) shown in bold. Abbreviations as in Supp. Table B1.

	MAPER				FreeSurfer			
**Region**	**Right Hemisphere**		**Left Hemisphere**		**Right Hemisphere**		**Left Hemisphere**	
	**% diff**	**p-value**	**% diff**	**p-value**	**% diff**	**p-value**	**% diff**	**p-value**
Temporal lobe								
hippocampus	**-17.69**	**1.46E-08**	**-22.68**	**9.44E-11**	**-20.67**	**1.98E-08**	**-25.59**	**7.31E-12**
amygdala	**-16.42**	**1.44E-05**	**-15.42**	**7.22E-05**	**-20.12**	**6.91E-07**	**-25.78**	**3.71E-09**
ant. temp. lobe, med.	-5.27	1.60E-01	-7.96	2.02E-02	-4.40	1.98E-01	-7.37	3.21E-02
ant. temp. lobe, lat.	-8.37	1.96E-02	-11.37	1.81E-02	-7.88	4.35E-02	-6.55	1.86E-01
parahippocampal gyr.	**-14.05**	**6.53E-06**	**-17.31**	**3.39E-08**	**-22.52**	**1.04E-08**	**-23.12**	**9.95E-09**
sup. temp. gyr., post.	-7.11	7.97E-03	-7.50	1.87E-02	-4.25	8.28E-02	-5.32	4.03E-02
middle & inf. temp. gyr.	-9.37	3.82E-03	**-11.98**	**1.68E-04**	-9.02	8.83E-04	-9.74	1.60E-03
fusiform gyr.	-6.25	9.65E-02	**-13.16**	**1.20E-04**	-10.47	6.38E-02	-13.00	8.23E-03
post. temp. lobe	**-8.56**	**3.29E-04**	-7.76	6.80E-04	**-9.46**	**3.72E-04**	**-10.48**	**1.87E-04**
sup. temp. gyr., ant.	-3.28	3.99E-01	-4.04	3.26E-01	-10.19	1.58E-03	-7.82	2.51E-02
Insula and cingulate gyri								
insula, post. long gyr.	-9.41	7.77E-03	-8.81	4.74E-03	-4.14	1.88E-01	-2.99	5.24E-01
insula, ant. short gyr.	-9.99	8.89E-03	-6.79	1.13E-01	-5.58	2.52E-01	-7.96	3.64E-02
insula, middle short gyr.	-8.11	1.14E-01	-9.78	4.72E-02	-6.22	3.62E-01	-2.04	8.01E-01
insula, post. short gyr.	-6.36	1.35E-01	-5.90	8.81E-02	-4.17	3.12E-01	0.34	8.04E-01
insula, ant. inf. cort.	-9.04	3.56E-02	-8.26	5.57E-02	-7.03	8.40E-02	-7.10	2.59E-02
insula, ant. long gyr.	-5.40	9.86E-02	-3.51	2.07E-01	-3.07	4.07E-01	2.54	4.41E-01
cingulate gyrus, ant.	-1.07	8.81E-01	0.41	7.78E-01	-2.08	6.21E-01	-1.04	8.48E-01
cingulate gyrus, post.	-3.28	2.55E-01	-5.79	3.84E-02	-3.57	1.80E-01	-7.09	9.95E-03
Frontal lobe								
middle frontal gyr.	-2.42	3.64E-01	-2.35	3.93E-01	-3.07	2.61E-01	-4.60	6.96E-02
precentral gyr.	-0.09	8.90E-01	-2.28	2.81E-01	-0.46	7.32E-01	-0.53	6.42E-01
ant. orbital gyr.	-2.62	3.16E-01	1.36	6.52E-01	-2.97	2.46E-01	-2.30	4.20E-01
inf. frontal gyr.	0.21	7.95E-01	-1.64	5.92E-01	1.27	6.18E-01	-3.42	2.24E-01
sup. frontal gyr.	-2.55	1.51E-01	-0.98	6.07E-01	-3.29	7.19E-02	-3.63	6.19E-02
med. orbital gyr.	-0.16	9.93E-01	0.23	9.73E-01	-2.44	2.81E-01	-1.20	5.29E-01

lat. orbital gyr.	-5.16	1.41E-01	-4.52	1.19E-01	-4.55	1.70E-01	-5.10	6.13E-02
post. orbital gyr.	-2.78	3.88E-01	-5.22	5.50E-02	0.38	8.53E-01	-3.12	3.27E-01
subgenual frontal cort.	-0.48	9.09E-01	6.48	1.12E-01	-3.91	5.20E-01	4.06	2.81E-01
pre-subgenual frontal cort.	-1.07	8.22E-01	13.46	2.70E-02	0.01	8.96E-01	10.25	6.33E-02
Occipital lobe								
lingual gyr.	-4.12	2.25E-01	-2.48	5.12E-01	-7.36	1.98E-02	-5.03	1.43E-01
cuneus	-7.86	4.97E-02	0.05	9.75E-01	-4.85	1.06E-01	-1.74	6.66E-01
occipital lobe	-8.17	3.66E-03	-8.89	5.93E-03	-6.18	1.52E-02	-6.72	9.24E-03
Parietal lobe								
straight gyr.	1.34	6.23E-01	0.12	9.17E-01	-4.15	9.33E-02	-2.69	3.01E-01
postcentral gyr.	-0.17	8.50E-01	-0.49	8.23E-01	1.53	5.86E-01	-1.30	6.00E-01
sup. parietal gyr.	-7.18	1.63E-02	-5.23	9.07E-02	-6.85	4.94E-03	-8.56	2.01E-03
supramarginal gyr.	-6.06	6.53E-02	-4.30	1.17E-01	-4.38	1.43E-01	-4.22	1.52E-01
angular gyr.	-10.75	6.54E-04	-9.49	6.09E-03	-11.62	1.45E-03	-8.50	3.49E-03
Central structures								
caudate nucleus	6.30	9.81E-02	4.02	2.28E-01	1.66	4.67E-01	1.43	5.23E-01
nucleus accumbens	-8.41	5.98E-02	-12.63	1.36E-02	-13.83	2.05E-02	-15.32	9.05E-03
putamen	-1.68	6.44E-01	-1.31	7.30E-01	-7.11	3.12E-02	-4.63	2.23E-01
thalamus	**-8.16**	**2.01E-05**	-5.73	7.07E-03	-6.31	1.08E-03	-6.38	1.56E-03
pallidum	-1.89	4.39E-01	-3.53	3.11E-01	-3.94	1.54E-01	-4.33	2.69E-01
corpus callosum (np)	-4.72	2.74E-02			0.99	4.41E-01		
substantia nigra	-6.70	1.17E-01	-6.62	9.13E-02	-9.90	3.56E-01	1.72	9.54E-01
Posterior fossa & ventricles								
cerebellum	-2.20	2.29E-01	-1.70	3.08E-01	-2.07	2.65E-01	-1.96	2.48E-01
brainstem (np)	-3.50	6.82E-02			-4.38	2.81E-02		
lat. ventricle, main	**37.03**	**2.16E-06**	**32.42**	**1.69E-05**	**44.99**	**2.02E-06**	**40.56**	**3.37E-06**
lat. ventricle, temp. horn	**36.40**	**1.42E-04**	**39.47**	**5.17E-05**	**30.50**	**2.80E-04**	**35.52**	**5.12E-05**
third ventricle (np)	14.50	1.44E-02			12.88	1.14E-02		

Supplementary Table D2. Group differences for the DKT40 atlas database.

Group differences between healthy control (HC) subjects and Alzheimer's Disease (AD) patients for the DKT40 atlas set with the MAPER and FreeSurfer segmentation methods. Negative percentage difference values indicate smaller volumes in AD than HC. Significant differences in structure volumes between HC and AD groups (from the ANCOVA, after adjustment for 62 multiple comparisons) shown in bold. Abbreviations as in Supp. Table B1.

	MAPER				FreeSurfer			
**Region**	**Right Hemisphere**		**Left Hemisphere**		**Right Hemisphere**		**Left Hemisphere**	
	**% diff**	**p-value**	**% diff**	**p-value**	**% diff**	**p-value**	**% diff**	**p-value**
Temporal lobe								
entorhinal cort.	**-17.28**	**3.12E-05**	**-19.84**	**3.65E-06**	**-26.09**	**1.70E-07**	**-27.03**	**5.32E-09**
parahippocampal gyr.	**-13.61**	**1.54E-04**	**-18.94**	**1.20E-06**	**-15.93**	**4.35E-06**	**-13.91**	**1.65E-04**
sup. temp. gyr.	-5.58	2.76E-02	-5.92	1.82E-02	-6.31	3.39E-03	-5.86	1.50E-02
middle temp. gyr.	**-8.40**	**5.65E-04**	**-9.80**	**1.09E-04**	-6.34	7.59E-03	**-9.26**	**7.61E-04**
inf. temp. gyr.	-9.31	2.28E-03	**-9.98**	**5.56E-04**	-10.48	1.98E-03	**-12.13**	**1.84E-04**
fusiform gyr.	-4.36	1.56E-01	-7.16	1.54E-02	**-11.60**	**4.63E-05**	**-11.74**	**2.98E-04**
transverse temp. gyr.	-4.88	1.98E-01	-2.47	5.79E-01	0.30	8.87E-01	-1.07	7.30E-01
Insula and cingulate gyri								
insula cort.	-6.28	5.42E-03	-5.24	1.28E-02	-4.70	5.17E-02	-3.54	9.79E-02
cingulate gyr., rostral ant.	-2.83	5.86E-01	4.65	2.07E-01	0.85	7.73E-01	0.47	7.97E-01
cingulate gyr., caudal ant.	2.24	5.02E-01	4.97	9.08E-02	-5.47	2.50E-01	-2.04	4.42E-01
cingulate gyr., post.	2.46	3.20E-01	1.77	4.69E-01	-2.98	3.40E-01	-4.78	9.81E-02
cingulate gyr., isthmus	-9.09	2.63E-02	**-15.51**	**6.28E-05**	-7.77	1.96E-02	**-12.36**	**1.33E-04**
Frontal lobe								
middle frontal gyr., rostral	-2.93	2.74E-01	-2.21	3.96E-01	-2.40	3.98E-01	-5.38	4.06E-02
middle frontal gyr., caudal	-1.80	5.77E-01	-5.33	1.18E-01	-3.50	4.05E-01	-2.84	3.69E-01
sup. frontal gyr.	-3.15	1.17E-01	-2.18	2.75E-01	-3.54	4.90E-02	-3.53	7.83E-02
pars opercularis	3.56	1.71E-01	4.42	1.82E-01	6.10	5.89E-02	-0.60	8.24E-01
pars triangularis	-4.49	2.51E-01	-5.09	2.24E-01	-3.76	4.86E-01	-5.00	1.53E-01
pars orbitalis	-3.07	5.13E-01	-3.97	4.66E-01	-2.12	4.97E-01	-6.67	5.52E-02
lat. orbitofrontal	0.54	7.59E-01	0.00	9.75E-01	-1.49	3.87E-01	-2.16	2.08E-01
med. orbitofrontal	0.63	6.67E-01	-3.00	2.02E-01	-4.57	6.08E-02	-2.05	4.04E-01
precentral gyr.	-0.78	6.59E-01	-2.01	3.68E-01	-1.15	5.37E-01	-0.88	5.49E-01
paracentral gyr.	0.21	9.26E-01	-3.19	2.13E-01	5.00	1.15E-01	-0.17	8.96E-01
Occipital lobe								

lingual gyr.	-3.32	2.98E-01	-2.33	5.38E-01	-7.82	1.19E-02	-5.70	9.28E-02
cuneus	-6.24	1.36E-01	0.19	9.26E-01	-5.63	5.49E-02	-4.24	2.17E-01
lat. occipital lobe	-9.47	3.32E-03	-11.69	5.63E-03	-3.80	2.61E-01	-4.48	1.78E-01
pericalcarine cort.	-0.85	9.18E-01	-0.23	9.64E-01	0.28	9.27E-01	4.18	2.94E-01
Parietal lobe								
postcentral gyr.	-0.03	9.68E-01	0.87	7.68E-01	0.39	9.18E-01	-1.27	6.45E-01
sup. parietal gyr.	-8.80	6.53E-03	-4.38	2.68E-01	-7.83	8.97E-03	-8.18	1.44E-02
supramarginal gyr.	-6.48	2.75E-02	-2.89	2.64E-01	-4.12	1.80E-01	-4.26	1.31E-01
inf. parietal gyr.	**-11.22**	**2.44E-04**	-8.59	8.66E-03	**-10.43**	**6.91E-04**	-8.19	5.06E-03
precuneus	-5.25	6.76E-02	-8.54	2.38E-03	-6.08	1.06E-02	**-9.49**	**3.26E-04**

Supplementary Table D3. Group differences for the MGC2012 atlas database.

Group differences between healthy control (HC) subjects and Alzheimer's Disease (AD) patients for the MGC2012 atlas set with the MAPER and FreeSurfer segmentation methods. Negative percentage difference values indicate smaller volumes in AD than HC. Significant differences in structure volumes between HC and AD groups (from the ANCOVA, after adjustment for 132 multiple comparisons) shown in bold. Abbreviations as in Supp. Table B1.

**Region**	**MAPER**				**FreeSurfer**			
	**Right Hemisphere**		**Left Hemisphere**		**Right Hemisphere**		**Left Hemisphere**	
	**% diff**	**p-value**	**% diff**	**p-value**	**% diff**	**p-value**	**% diff**	**p-value**
Temporal lobe								
hippocampus	**-9.65**	**2.89E-04**	**-10.78**	**1.24E-04**	**-20.00**	**4.16E-10**	**-22.88**	**2.54E-11**
amygdala	**-17.04**	**9.45E-06**	**-18.24**	**3.07E-06**	**-34.46**	**9.82E-07**	**-37.26**	**4.86E-08**
entorhinal area	**-13.14**	**2.34E-04**	**-16.12**	**3.70E-05**	-21.08	2.34E-03	**-26.30**	**1.20E-06**
parahippocampal gyr.	**-10.07**	**2.99E-04**	**-13.42**	**5.99E-06**	**-20.79**	**1.42E-08**	**-19.88**	**2.68E-08**
sup. temp. gyr.	-6.28	2.15E-02	-7.60	1.04E-02	-7.98	7.33E-03	-6.31	4.93E-02
middle temp. gyr.	-5.49	7.42E-03	-6.43	4.18E-03	-6.51	6.57E-03	-8.42	2.20E-03
inf. temp. gyr.	-7.12	8.60E-03	-8.18	2.05E-03	-10.56	2.47E-03	**-12.63**	**1.87E-04**
fusiform gyr.	-3.48	2.37E-01	-4.93	1.11E-01	-11.18	5.12E-04	-11.29	6.46E-04
transverse temp. gyr.	-1.19	6.59E-01	2.73	5.49E-01	-0.04	7.96E-01	-1.18	6.88E-01
temporal pole	-2.93	3.64E-01	-3.15	3.91E-01	-6.27	1.45E-02	-6.54	3.03E-02
planum temporale	-1.37	7.07E-01	-3.01	5.58E-01	-4.63	2.31E-01	-7.38	5.72E-02
planum polare	1.56	5.58E-01	-1.52	6.03E-01	-3.51	2.43E-01	-3.83	1.99E-01
Insula and cingulate gyri								
ant. insula	-5.15	5.43E-02	-3.63	2.05E-01	-6.28	2.51E-02	-4.92	5.90E-02
post. insula	-2.52	3.44E-01	-2.24	3.99E-01	-3.21	2.51E-01	0.39	8.58E-01
cingulate gyrus, ant.	-0.20	8.85E-01	3.02	2.01E-01	-0.69	9.36E-01	-1.67	6.69E-01
cingulate gyrus, middle	4.30	4.08E-02	8.66	4.39E-04	-3.16	3.80E-01	-2.10	5.33E-01
cingulate gyrus, post.	-6.30	2.54E-02	**-9.47**	**1.52E-04**	-7.24	1.05E-02	**-10.20**	**2.66E-04**
Frontal lobe								
middle frontal gyr.	-0.84	8.35E-01	-1.73	4.74E-01	-2.47	3.90E-01	-4.00	1.17E-01
precentral gyr.	0.00	9.84E-01	-1.04	6.83E-01	-1.82	4.00E-01	-1.76	3.26E-01
precentral gyr., med.	0.13	9.90E-01	-2.37	4.50E-01	5.40	1.48E-01	0.44	9.88E-01
sup. frontal gyr.	-4.09	1.30E-01	-0.06	9.86E-01	-5.12	2.86E-02	-2.81	2.24E-01
sup. frontal gyr., med.	1.51	3.87E-01	1.01	5.35E-01	-3.81	1.13E-01	-4.41	1.22E-01
med. frontal cort.	3.82	1.00E-01	1.69	6.12E-01	-5.62	1.12E-01	-2.28	5.28E-01

inf. frontal gyr., opercular	5.99	8.01E-02	4.55	1.84E-01	5.98	1.41E-01	-1.28	7.23E-01
inf. frontal gyr., orbital	1.32	7.58E-01	-2.12	8.36E-01	1.40	6.98E-01	-3.88	3.80E-01
inf. frontal gyr., triangular	-4.42	1.81E-01	-2.43	7.09E-01	-4.18	4.25E-01	-4.86	1.94E-01
frontal operculum	-0.53	8.67E-01	-2.78	5.09E-01	3.92	3.06E-01	0.41	9.76E-01
frontal pole	0.45	9.05E-01	-3.23	4.93E-01	-3.22	3.56E-01	-3.72	2.96E-01
central operculum	2.99	2.06E-01	4.29	3.96E-02	1.98	3.96E-01	2.18	4.28E-01
ant. orbital gyr.	1.69	6.11E-01	0.96	7.47E-01	-2.30	4.07E-01	-4.27	3.00E-01
post. orbital gyr.	1.67	5.43E-01	-1.17	8.65E-01	0.07	9.76E-01	-4.86	1.15E-01
med. orbital gyr.	1.31	5.72E-01	1.33	6.42E-01	-2.14	3.34E-01	0.38	9.50E-01
lat. orbital gyr.	-6.47	9.79E-02	-1.24	7.66E-01	-5.64	1.18E-01	-7.35	1.58E-02
supplementary motor cort.	2.68	2.90E-01	-3.69	1.37E-01	2.98	2.67E-01	-3.32	1.77E-01
Occipital lobe								
lingual gyr.	-2.30	3.99E-01	-1.84	6.28E-01	-8.36	6.53E-03	-6.48	5.56E-02
cuneus	-6.11	6.78E-02	1.26	5.76E-01	-4.78	1.18E-01	-4.87	1.83E-01
sup. occipital gyr.	-9.27	6.51E-03	-7.44	3.07E-02	-8.60	3.57E-02	-10.79	4.30E-03
middle occipital gyr.	-8.47	1.43E-02	-7.73	4.28E-02	-6.56	1.37E-01	-6.11	1.09E-01
inf. occipital gyr.	-4.41	1.10E-01	-7.36	2.96E-02	-4.94	2.11E-01	-6.63	8.38E-02
occipital pole	-2.76	6.31E-01	-7.47	1.97E-01	-2.48	5.90E-01	0.05	9.54E-01
occipital fusiform gyr.	-5.09	9.78E-02	-10.51	4.72E-03	-8.94	2.12E-02	-8.24	2.72E-02
calcarine cort.	-1.48	9.57E-01	2.19	3.98E-01	-0.57	8.97E-01	3.05	3.59E-01
Parietal lobe								
gyrus rectus	4.99	1.49E-01	1.40	7.40E-01	-3.76	1.48E-01	-1.40	5.90E-01
postcentral gyr.	0.78	7.18E-01	-0.31	9.66E-01	-0.21	8.97E-01	-2.31	4.48E-01
postcentral gyr., med.	-0.10	9.76E-01	-3.37	4.95E-01	2.83	5.13E-01	-2.77	5.25E-01
sup. parietal lobule	-7.19	1.10E-02	-2.73	4.47E-01	-7.17	2.00E-02	-8.01	2.87E-02
supramarginal gyr.	-5.12	1.48E-01	-3.88	1.97E-01	-5.95	7.61E-02	-5.74	4.79E-02
precuneus	-3.19	2.72E-01	-6.35	9.88E-03	-6.56	8.85E-03	**-9.47**	**3.24E-04**
parietal operculum	0.34	9.39E-01	4.75	1.53E-01	2.64	5.89E-01	2.74	4.86E-01
angular gyrus	-7.90	8.31E-03	-5.68	8.81E-02	-11.78	9.07E-04	-8.83	3.79E-03
Central structures								
caudate	6.66	1.35E-01	4.18	2.32E-01	-0.36	9.37E-01	-3.39	4.37E-01
accumbens area	-5.00	2.63E-01	-11.76	3.03E-02	-16.65	4.75E-02	-28.31	1.72E-02
putamen	-2.75	4.54E-01	-2.19	5.98E-01	-9.68	1.14E-02	-8.48	5.81E-02
thalamus (proper)	**-7.78**	**1.97E-05**	-6.82	1.09E-03	**-6.37**	**1.88E-04**	**-5.87**	**1.53E-04**
pallidum	-0.83	6.70E-01	-1.71	5.93E-01	-2.99	1.78E-01	-0.56	7.27E-01

ventral diencephalon	-4.49	5.83E-03	-5.99	7.60E-04	-4.96	6.08E-03	-7.05	6.65E-04
optic chiasm (np)	6.34	2.50E-02			-5.08	6.76E-01		
subcallosal area	0.99	6.06E-01	-0.82	9.45E-01	-7.02	1.37E-01	-5.44	2.09E-01
Posterior fossa, ventricles & white matter								
cerebellum exterior	-1.83	3.33E-01	-1.21	5.16E-01	-1.52	4.91E-01	-1.53	4.76E-01
cerebellar vermal lobules I-V (np)	-4.72	5.28E-02			-5.41	9.31E-02		
cerebellar vermal lobules VI-VII (np)	-5.40	4.54E-02			-3.07	1.74E-01		
cerebellar vermal lobules VIII-X (np)	-0.13	9.81E-01			-2.85	2.78E-01		
brainstem (np)	-4.02	4.61E-02			-4.31	3.75E-02		
lateral ventricle	**46.22**	**1.36E-06**	**47.44**	**7.03E-06**	**47.30**	**1.16E-06**	**41.77**	**4.38E-06**
inf. lateral ventricle	**45.56**	**1.38E-04**	**48.09**	**1.24E-05**	**69.58**	**3.07E-06**	**73.70**	**4.74E-07**
third ventricle (np)	19.81	4.66E-03			13.54	1.28E-02		
fourth ventricle (np)	4.35	4.08E-01			9.75	1.14E-01		
cerebrospinal fluid (np)	6.29	1.97E-01			14.52	3.78E-03		
cerebral white matter	-3.56	3.15E-02	-3.91	2.41E-02	-4.71	3.77E-04	-4.40	2.41E-03
cerebellar white matter	-3.56	2.76E-01	-4.32	1.63E-01	-1.73	4.64E-01	-1.84	3.74E-01

Supplementary Table E1. Group differences for the HM atlas database (left HS).

Group differences between healthy control (HC) subjects and left hippocampal sclerosis (LHS) patients for the HM atlas set with the MAPER and FreeSurfer segmentation methods. Negative percentage difference values indicate smaller volumes in LHS than HC. Significant differences in structure volumes between HC and LHS groups (from the ANCOVA, after adjustment for 93 multiple comparisons) shown in bold. Abbreviations as in Supp. Table B1.

	**MAPER**				**FreeSurfer**			
**Region**	**Right Hemisphere**		**Left Hemisphere**		**Right Hemisphere**		**Left Hemisphere**	
	**% diff**	**p-value**	**% diff**	**p-value**	**% diff**	**p-value**	**% diff**	**p-value**
Temporal lobe								
hippocampus	-4.39	1.24E-01	**-32.52**	**7.00E-20**	-4.63	2.14E-01	**-34.15**	**8.00E-20**
amygdala	-7.68	5.53E-03	-1.89	4.74E-01	-1.62	6.48E-01	-8.46	2.12E-02
ant. temp. lobe, med.	-5.18	2.54E-02	-8.95	2.78E-03	-4.17	2.08E-01	**-15.64**	**2.54E-05**
ant. temp. lobe, lat.	-2.25	3.63E-01	-4.07	2.29E-01	-3.99	2.94E-01	-12.33	6.11E-03
parahippocampal gyr.	-6.97	6.48E-03	-5.55	1.88E-02	3.45	2.11E-01	**-11.25**	**2.13E-04**
sup. temp. gyr., post.	-1.31	6.54E-01	-3.65	1.32E-01	-4.55	8.13E-02	-7.62	6.28E-03
middle & inf. temp. gyr.	-3.99	3.72E-02	-0.79	8.28E-01	-0.96	8.28E-01	-6.09	1.38E-02
fusiform gyr.	-5.87	1.73E-02	-1.77	4.90E-01	-1.58	7.37E-01	-9.14	4.11E-02
post. temp. lobe	-0.07	9.72E-01	-4.73	3.05E-03	-3.79	1.47E-01	**-8.73**	**3.58E-04**
sup. temp. gyr., ant.	-5.32	1.03E-01	-9.67	5.01E-03	-7.12	5.60E-02	**-16.82**	**1.14E-06**
Insula and cingulate gyri								
insula, post. long gyr.	-4.79	4.94E-02	-1.31	6.65E-01	3.78	1.89E-01	-2.01	5.62E-01
insula, ant. short gyr.	-1.61	6.39E-01	-0.25	8.50E-01	0.76	7.80E-01	-0.74	9.49E-01
insula, middle short gyr.	-1.44	6.37E-01	-0.94	6.93E-01	-7.48	7.63E-02	-3.59	3.47E-01
insula, post. short gyr.	-2.69	4.07E-01	-1.39	7.30E-01	-4.09	2.16E-01	-10.24	4.78E-03
insula, ant. inf. cort.	-3.77	1.76E-01	-0.41	9.94E-01	-7.02	1.29E-01	-13.43	1.07E-03
insula, ant. long gyr.	-4.49	7.35E-02	-1.80	4.55E-01	-5.22	1.06E-01	-9.27	9.22E-03
cingulate gyrus, ant.	-4.19	2.17E-01	-1.01	7.66E-01	1.02	6.74E-01	-2.78	3.88E-01
cingulate gyrus, post.	-0.51	8.27E-01	-1.74	5.51E-01	-2.44	3.12E-01	-1.41	7.31E-01
Frontal lobe								
middle frontal gyr.	-0.34	9.17E-01	-3.14	1.15E-01	-2.86	3.34E-01	-7.79	1.64E-03
precentral gyr.	-1.76	4.44E-01	-1.02	6.38E-01	-6.72	1.83E-02	-6.38	2.84E-02
ant. orbital gyr.	-2.86	1.86E-01	-1.96	2.69E-01	0.60	6.48E-01	-0.02	8.55E-01
inf. frontal gyr.	-3.39	1.22E-01	-2.23	4.11E-01	-4.90	1.12E-01	-3.57	3.11E-01
sup. frontal gyr.	-7.19	1.90E-03	-5.01	2.09E-02	-6.35	8.60E-03	-5.80	1.99E-02

med. orbital gyr.	-0.94	6.84E-01	-0.60	6.55E-01	-1.01	8.52E-01	-2.53	3.55E-01
lat. orbital gyr.	-1.96	5.57E-01	-0.75	8.92E-01	-4.83	1.62E-01	-5.87	8.51E-02
post. orbital gyr.	-1.85	3.61E-01	-3.92	7.10E-02	-2.90	3.51E-01	-2.76	3.63E-01
subgenual frontal cort.	-1.38	6.74E-01	-6.08	8.01E-02	-6.31	2.36E-01	-1.44	7.80E-01
pre-subgenual frontal cort.	-6.76	1.98E-01	-4.49	3.04E-01	-7.22	1.37E-01	0.23	8.46E-01
Occipital lobe								
lingual gyr.	-0.36	9.23E-01	-1.06	7.11E-01	-0.25	9.47E-01	-6.08	1.30E-01
cuneus	-3.20	3.37E-01	-6.14	1.13E-01	-7.59	9.34E-03	-7.15	2.31E-02
occipital lobe	-1.51	4.99E-01	-2.14	3.53E-01	-3.82	1.38E-01	-6.03	4.11E-02
Parietal lobe								
straight gyr.	-0.53	7.77E-01	-0.11	9.93E-01	-3.19	2.54E-01	-1.53	6.64E-01
postcentral gyr.	-5.41	9.56E-03	-4.20	5.01E-02	**-10.59**	**2.70E-05**	**-8.49**	**9.88E-04**
sup. parietal gyr.	-2.66	2.29E-01	-2.87	1.77E-01	-4.15	1.07E-01	-4.36	9.82E-02
supramarginal gyr.	-5.45	2.13E-02	-2.70	2.99E-01	-4.63	1.34E-01	-4.97	1.05E-01
angular gyr.	-1.12	8.02E-01	-7.80	1.57E-02	-5.48	1.54E-01	-8.39	8.20E-03
Central structures								
caudate nucleus	-2.53	3.18E-01	-0.23	9.75E-01	-2.96	4.66E-01	-1.22	7.14E-01
nucleus accumbens	-3.71	3.24E-01	-4.09	1.83E-01	-42.57	5.02E-02	-26.56	8.17E-02
putamen	-2.93	1.30E-01	-5.08	7.42E-03	-1.48	4.64E-01	-4.86	1.37E-02
thalamus	-5.07	5.06E-02	-8.17	7.10E-04	-5.28	3.10E-03	**-9.39**	**1.80E-06**
pallidum	-5.16	2.87E-01	-8.19	3.11E-02	-0.04	9.44E-01	0.82	8.30E-01
corpus callosum (np)	-1.49	5.23E-01			2.89	3.50E-01		
substantia nigra	-8.46	1.94E-03	-8.32	1.77E-03	-30.25	1.25E-01	-21.37	1.53E-01
Posterior fossa & ventricles								
cerebellum	-1.06	5.50E-01	-1.00	5.59E-01	-0.12	9.87E-01	1.53	5.15E-01
brainstem (np)	-0.52	7.64E-01			0.82	7.07E-01		
lat. ventricle, main	-16.64	2.15E-02	-20.94	1.64E-02	21.50	2.33E-02	24.83	1.53E-02
lat. ventricle, temp. horn	-6.81	3.33E-01	-7.26	7.89E-02	-4.00	5.97E-01	7.42	2.34E-01
third ventricle (np)	**-21.87**	**4.03E-04**			27.26	7.41E-04		

Supplementary Table E2. Group differences for the DKT40 atlas database (left HS).

Group differences between healthy control (HC) subjects and left hippocampal sclerosis (LHS) patients for the DKT40 atlas set with the MAPER and FreeSurfer segmentation methods. Negative percentage difference values indicate smaller volumes in LHS than HC. Significant differences in structure volumes between HC and LHS groups (from the ANCOVA, after adjustment for 62 multiple comparisons) shown in bold. Abbreviations as in Supp. Table B1.

Region	MAPER				FreeSurfer			
	Right Hemisphere		Left Hemisphere		Right Hemisphere		Left Hemisphere	
	% diff	p-value	% diff	p-value	% diff	p-value	% diff	p-value
Temporal lobe								
entorhinal cort.	6.04	6.34E-02	-8.91	5.26E-03	6.03	1.37E-01	-14.62	1.04E-03
parahippocampal gyr.	6.41	1.72E-02	-4.13	1.62E-01	5.61	4.18E-02	-7.02	5.53E-02
sup. temp. gyr.	0.65	6.05E-01	-3.4	2.89E-02	-4.92	4.70E-02	**-12.22**	**2.64E-06**
middle temp. gyr.	0.69	6.07E-01	-6.16	5.59E-03	-3.16	2.53E-01	-8.05	1.73E-03
inf. temp. gyr.	2.4	1.95E-01	-2.23	2.62E-01	-1.99	5.26E-01	-8.29	9.68E-04
fusiform gyr.	-0.56	8.62E-01	-1.01	6.83E-01	-4.5	9.00E-02	-7.05	1.97E-02
transverse temp. gyr.	0.82	7.55E-01	4.06	3.88E-01	0.03	8.19E-01	-4.64	2.95E-01
Insula and cingulate gyri								
insula cort.	1.6	2.50E-01	-0.39	7.08E-01	-2.61	3.14E-01	-6.73	7.95E-03
cingulate gyr., rostral ant.	-4.18	1.96E-01	-0.69	8.86E-01	-3.89	3.05E-01	-0.77	9.15E-01
cingulate gyr., caudal ant.	1.4	5.14E-01	-3.12	3.06E-01	2.84	4.21E-01	-3.95	1.48E-01
cingulate gyr., post.	-1.08	8.73E-01	-2.55	6.20E-01	-3.21	2.10E-01	-5.37	3.76E-02
cingulate gyr., isthmus	4.24	2.48E-01	-0.13	9.14E-01	2.28	3.85E-01	2.64	2.85E-01
Frontal lobe								
middle frontal gyr., rostral	-2.42	4.33E-01	-2.39	3.74E-01	-5.92	5.80E-02	**-9.95**	**2.21E-04**
middle frontal gyr., caudal	0.33	7.91E-01	0.23	7.83E-01	2.69	2.71E-01	-3.83	3.22E-01
sup. frontal gyr.	-2.78	1.43E-01	-1.64	4.36E-01	-6.27	6.34E-03	-5.87	1.91E-02
pars opercularis	-2.11	4.60E-01	-1.98	4.69E-01	-4.64	2.04E-01	-4.74	2.26E-01
pars triangularis	1.53	5.51E-01	4.94	7.36E-02	-4.37	3.44E-01	-1.4	8.90E-01
pars orbitalis	-3.5	2.31E-01	-2.51	4.16E-01	-6.47	3.53E-02	-5.52	4.63E-02
lat. orbitofrontal	1.48	2.92E-01	-1.38	3.83E-01	0.11	7.48E-01	-2.96	1.48E-01
med. orbitofrontal	-1.5	4.36E-01	0.75	6.37E-01	-3.43	1.94E-01	-0.66	9.69E-01
precentral gyr.	-0.31	8.85E-01	-0.58	7.37E-01	-7.4	1.02E-02	-7.33	1.08E-02
paracentral gyr.	-3.32	1.83E-01	-2.2	3.97E-01	-4.84	1.44E-01	-1.22	8.49E-01
Occipital lobe								

lingual gyr.	1.5	4.20E-01	-0.03	9.82E-01	-0.12	9.10E-01	-7.22	7.08E-02
cuneus	-4.81	1.04E-01	-3.98	1.90E-01	-6.62	2.52E-02	-6.68	6.47E-02
lat. occipital lobe	1.35	6.57E-01	-2.01	5.39E-01	-2.36	4.73E-01	-4.19	2.12E-01
pericalcarine cort.	-6.49	1.65E-01	-4.47	3.92E-01	-9.33	1.18E-01	-6.53	1.90E-01
Parietal lobe								
postcentral gyr.	-5.81	5.79E-03	-2.22	2.52E-01	**-11.89**	**3.79E-05**	-8.9	1.82E-03
sup. parietal gyr.	2.91	1.02E-01	1.47	4.83E-01	-2.71	4.49E-01	-3.54	2.28E-01
supramarginal gyr.	-4.34	4.40E-02	-1.67	5.11E-01	-4.62	1.51E-01	-5.28	9.23E-02
inf. parietal gyr.	-1.56	5.32E-01	-3.97	1.15E-01	-5.98	5.68E-02	-9.13	1.86E-03
precuneus	-2.67	1.69E-01	-3.73	6.77E-02	-5.57	2.21E-02	-5.13	7.20E-02

Supplementary Table E3. Group differences for the MGC2012 atlas database (left HS).

Group differences between healthy control (HC) subjects and left hippocampal sclerosis (LHS) patients for the MGC2012 atlas set with the MAPER and FreeSurfer segmentation methods. Negative percentage difference values indicate smaller volumes in LHS than HC. Significant differences in structure volumes between HC and LHS groups (from the ANCOVA, after adjustment for 132 multiple comparisons) shown in bold. Abbreviations as in Supp. Table B1.

Region	MAPER				FreeSurfer			
	Right Hemisphere		Left Hemisphere		Right Hemisphere		Left Hemisphere	
	% diff	p-value	% diff	p-value	% diff	p-value	% diff	p-value
Temporal lobe								
hippocampus	-4.80	3.18E-02	**-23.01**	**1.95E-17**	-5.10	1.53E-01	**-29.67**	**4.35E-21**
amygdala	-7.98	7.71E-03	-2.20	4.58E-01	-6.29	1.75E-01	-6.89	5.26E-02
entorhinal area	-6.95	2.03E-02	-7.40	1.92E-02	0.52	8.43E-01	**-18.73**	**2.08E-04**
parahippocampal gyr.	-6.94	1.63E-03	-1.32	4.88E-01	2.94	2.69E-01	**-11.62**	**2.88E-05**
sup. temp. gyr.	-1.71	4.06E-01	-1.36	5.62E-01	-5.63	3.89E-02	-10.01	1.05E-03
middle temp. gyr.	-1.67	3.50E-01	-4.49	4.07E-02	-2.99	2.81E-01	-7.69	3.07E-03
inf. temp. gyr.	-1.91	2.94E-01	-0.99	6.22E-01	-1.86	5.72E-01	-7.92	2.13E-03
fusiform gyr.	-0.98	6.19E-01	-1.51	5.11E-01	-4.90	6.83E-02	-6.80	2.67E-02
transverse temp. gyr.	-0.73	7.93E-01	-5.34	1.63E-01	0.02	8.29E-01	-4.40	3.03E-01
temporal pole	-2.50	2.28E-01	-3.36	1.27E-01	-4.71	1.44E-01	**-16.85**	**4.07E-07**
planum temporale	-3.76	2.39E-01	-0.38	8.73E-01	-4.86	2.31E-01	-5.00	2.28E-01
planum polare	-1.18	6.10E-01	-6.80	1.17E-02	-3.66	2.10E-01	-9.40	9.31E-04
Insula and cingulate gyri								
ant. insula	-2.23	2.79E-01	-1.81	4.19E-01	-4.59	1.11E-01	-7.62	6.42E-03
post. insula	-1.37	4.80E-01	-3.39	7.78E-02	-0.66	9.32E-01	-6.38	3.68E-02
cingulate gyrus, ant.	-1.07	7.45E-01	-0.59	8.50E-01	-2.74	4.83E-01	-0.10	9.09E-01
cingulate gyrus, middle	-5.35	4.53E-02	-0.22	9.28E-01	-0.44	9.40E-01	-6.24	3.14E-02
cingulate gyrus, post.	-2.55	2.25E-01	-3.47	1.05E-01	0.58	7.06E-01	0.43	6.97E-01
Frontal lobe								
middle frontal gyr.	-0.78	5.49E-01	-0.43	9.63E-01	-2.84	3.91E-01	-8.40	1.35E-03
precentral gyr.	-2.20	1.99E-01	-1.14	4.85E-01	-6.94	2.07E-02	-7.34	1.68E-02
precentral gyr., med.	-0.13	9.83E-01	-0.38	9.03E-01	-4.03	2.59E-01	-0.77	9.53E-01
sup. frontal gyr.	-2.63	2.40E-01	-0.56	7.71E-01	-7.03	9.55E-03	-4.45	1.39E-01
sup. frontal gyr., med.	-3.19	1.93E-01	-4.63	8.42E-02	-5.19	6.05E-02	-8.16	4.68E-03
med. frontal cort.	-1.19	7.31E-01	-4.07	2.15E-01	-1.83	6.08E-01	-0.08	9.08E-01

inf. frontal gyr., opercular	-2.11	5.50E-01	-1.06	7.74E-01	-5.63	2.07E-01	-4.65	3.12E-01
inf. frontal gyr., orbital	-6.11	1.19E-01	-2.06	5.47E-01	-5.38	1.33E-01	-7.43	9.55E-03
inf. frontal gyr., triangular	-1.97	5.14E-01	-7.55	1.10E-02	-5.48	2.35E-01	-2.44	6.64E-01
frontal operculum	-1.22	7.16E-01	-0.87	7.99E-01	-0.19	9.07E-01	-2.40	6.11E-01
frontal pole	-1.85	5.42E-01	-0.54	8.86E-01	-9.47	1.51E-02	-6.73	1.51E-01
central operculum	-0.58	7.45E-01	-0.18	8.81E-01	-5.89	3.09E-02	-7.64	8.55E-03
ant. orbital gyr.	-5.80	2.27E-02	-2.79	2.68E-01	0.18	8.17E-01	-2.91	4.01E-01
post. orbital gyr.	-0.18	9.32E-01	-3.41	1.35E-01	-2.10	5.57E-01	-3.81	2.00E-01
med. orbital gyr.	-0.47	7.72E-01	-1.79	3.61E-01	0.93	5.01E-01	-1.62	5.35E-01
lat. orbital gyr.	-1.86	4.71E-01	-2.59	3.68E-01	-5.34	1.40E-01	0.02	7.89E-01
supplementary motor cort.	-4.26	1.19E-01	-1.71	5.85E-01	-4.67	1.41E-01	-5.41	6.13E-02
Occipital lobe								
lingual gyr.	-2.75	2.61E-01	-0.95	7.71E-01	0.78	6.58E-01	-7.42	5.47E-02
cuneus	-2.80	3.67E-01	-3.49	2.61E-01	-6.04	3.73E-02	-7.04	5.10E-02
sup. occipital gyr.	-1.33	6.48E-01	-1.56	6.22E-01	-1.66	7.81E-01	-4.04	3.36E-01
middle occipital gyr.	-0.80	8.01E-01	-2.13	4.63E-01	-7.91	5.48E-02	-11.56	2.92E-03
inf. occipital gyr.	-0.72	8.38E-01	-3.80	2.42E-01	-5.02	1.85E-01	-5.08	1.75E-01
occipital pole	-2.53	4.40E-01	-3.42	3.16E-01	-3.93	2.38E-01	-0.10	9.64E-01
occipital fusiform gyr.	-6.89	1.18E-02	-2.76	2.93E-01	-0.16	9.46E-01	-6.43	2.72E-01
calcarine cort.	-1.34	7.06E-01	-1.46	7.13E-01	-7.85	1.81E-01	-3.54	4.76E-01
Parietal lobe								
gyrus rectus	-1.20	6.31E-01	-2.24	3.13E-01	-2.45	5.17E-01	-4.19	2.13E-01
postcentral gyr.	-4.08	3.49E-02	-1.39	4.92E-01	**-13.03**	**3.69E-05**	-8.90	3.26E-03
postcentral gyr., med.	-8.65	9.94E-02	-0.86	8.29E-01	-9.55	2.71E-02	-5.67	1.92E-01
sup. parietal lobule	-3.63	4.28E-02	-4.58	2.63E-02	-2.30	5.59E-01	-3.81	2.06E-01
supramarginal gyr.	-5.50	4.70E-02	-0.74	8.57E-01	-6.44	7.05E-02	-4.56	2.01E-01
precuneus	-1.25	5.88E-01	-2.54	2.67E-01	-5.18	4.19E-02	-5.21	7.13E-02
parietal operculum	-1.85	5.37E-01	-3.35	1.91E-01	-1.49	7.37E-01	-5.87	1.40E-01
angular gyrus	-0.63	8.14E-01	-2.97	2.39E-01	-4.94	1.83E-01	-8.19	8.88E-03
Central structures								
caudate	-2.03	5.30E-01	-1.00	6.03E-01	-4.05	4.03E-01	-3.10	2.94E-01
accumbens area	-1.90	6.23E-01	-2.65	4.30E-01	-2.47	5.82E-01	-6.90	1.06E-01
putamen	-0.66	8.32E-01	-2.72	2.51E-01	-1.39	5.01E-01	-5.07	1.12E-02
thalamus (proper)	-7.18	3.16E-03	**-10.23**	**1.67E-05**	-4.18	1.08E-02	**-7.12**	**1.22E-04**
pallidum	-0.28	8.73E-01	-1.05	6.48E-01	-0.42	8.18E-01	-1.37	5.89E-01

ventral diencephalon	-1.16	3.61E-01	-2.97	2.22E-02	-3.42	3.52E-02	-3.78	1.54E-02
optic chiasm (np)	-4.42	9.27E-02			-10.80	1.13E-01		
subcallosal area	-0.82	7.99E-01	-0.35	8.37E-01	-1.49	7.30E-01	-1.81	7.46E-01
Posterior fossa, ventricles & white matter								
cerebellum exterior	-1.24	4.72E-01	-1.26	4.59E-01	2.32	4.55E-01	4.69	1.58E-01
cerebellar vermal lobules I-V (np)	-2.32	4.16E-01			-0.15	9.90E-01		
cerebellar vermal lobules VI-VII (np)	-0.80	8.82E-01			-3.81	4.44E-01		
cerebellar vermal lobules VIII-X (np)	-0.61	8.59E-01			5.99	2.09E-01		
brainstem (np)	-0.23	9.31E-01			0.22	9.49E-01		
lateral ventricle	-18.82	2.90E-02	-22.66	1.67E-02	21.38	2.34E-02	24.41	1.36E-02
inf. lateral ventricle	-10.72	2.85E-01	-18.76	2.38E-03	6.13	5.06E-01	13.65	2.04E-02
third ventricle (np)	**-26.03**	**1.90E-04**			23.40	1.45E-03		
fourth ventricle (np)	-6.82	1.73E-01			6.44	2.55E-01		
cerebrospinal fluid (np)	-12.52	9.92E-02			20.64	3.91E-03		
cerebral white matter	-2.24	8.78E-02	-4.55	4.29E-04	3.17	1.98E-01	1.67	6.38E-01
cerebellar white matter	-5.68	9.87E-02	-4.79	1.24E-01	-3.20	6.32E-01	-0.79	8.98E-01

Supplementary Table E4. Group differences for the HM atlas database (right HS).

Group differences between healthy control (HC) subjects and right hippocampal sclerosis (RHS) patients for the HM atlas set with the MAPER and FreeSurfer segmentation methods. Negative percentage difference values indicate smaller volumes in RHS than HC. Significant differences in structure volumes between HC and RHS groups (from the ANCOVA, after adjustment for 93 multiple comparisons) shown in bold. Abbreviations as in Supp. Table B1.

Region	MAPER				FreeSurfer			
	Right Hemisphere		Left Hemisphere		Right Hemisphere		Left Hemisphere	
	% diff	p-value	% diff	p-value	% diff	p-value	% diff	p-value
Temporal lobe								
hippocampus	**-36.23**	**1.67E-15**	-3.95	9.60E-02	**-38.12**	**6.70E-12**	1.69	4.50E-01
amygdala	-2.05	8.64E-01	-7.26	1.35E-02	-12.78	9.81E-03	3.17	3.54E-01
ant. temp. lobe, med.	-2.40	2.45E-01	-7.08	6.00E-02	-11.91	7.52E-04	-2.41	3.76E-01
ant. temp. lobe, lat.	-4.23	2.41E-01	-3.87	2.93E-01	-2.60	5.62E-01	2.37	6.92E-01
parahippocampal gyr.	-3.61	2.96E-01	-6.79	1.20E-02	-10.78	4.02E-03	-3.15	3.02E-01
sup. temp. gyr., post.	-1.93	9.18E-01	-0.45	6.09E-01	-2.90	4.23E-01	-1.78	7.62E-01
middle & inf. temp. gyr.	-3.06	3.52E-01	-4.47	5.55E-02	-3.67	2.83E-01	0.40	7.79E-01
fusiform gyr.	-1.17	9.18E-01	-8.78	3.50E-03	-5.73	3.14E-01	-0.42	9.98E-01
post. temp. lobe	-4.49	4.45E-02	-0.17	6.77E-01	-5.06	2.41E-02	-3.54	8.41E-02
sup. temp. gyr., ant.	-9.69	2.78E-02	-4.00	2.03E-01	-13.89	1.10E-03	0.12	9.45E-01
Insula and cingulate gyri								
insula, post. long gyr.	-0.31	2.73E-01	-4.49	1.35E-02	8.82	1.06E-02	0.11	8.18E-01
insula, ant. short gyr.	-2.43	1.64E-01	-1.50	2.42E-01	2.32	5.10E-01	2.21	5.94E-01
insula, middle short gyr.	-13.57	7.86E-03	-5.72	3.82E-01	-5.09	3.10E-01	2.75	4.94E-01
insula, post. short gyr.	-3.50	9.75E-01	-3.70	9.37E-01	-0.02	9.36E-01	-4.22	3.18E-01
insula, ant. inf. cort.	-3.01	8.23E-01	-5.09	4.99E-02	-8.09	3.02E-02	-1.93	3.58E-01
insula, ant. long gyr.	-3.36	9.39E-01	-5.80	4.29E-01	-2.90	3.72E-01	-3.38	5.38E-01
cingulate gyrus, ant.	-0.08	7.90E-01	-1.02	9.09E-01	-1.19	9.71E-01	-2.53	6.52E-01
cingulate gyrus, post.	-0.53	5.97E-01	-2.93	7.25E-01	-2.95	2.20E-01	0.01	9.63E-01
Frontal lobe								
middle frontal gyr.	-2.89	7.61E-01	-1.41	6.89E-01	-2.74	3.44E-01	-0.57	8.70E-01
precentral gyr.	-4.90	4.35E-01	-4.56	4.69E-01	-6.43	3.02E-02	-5.74	4.40E-02
ant. orbital gyr.	-3.94	6.63E-02	-3.69	4.31E-02	1.25	5.96E-01	0.44	7.25E-01
inf. frontal gyr.	-0.52	2.90E-01	-1.66	8.95E-01	-3.27	3.01E-01	-1.34	7.63E-01
sup. frontal gyr.	-2.38	3.71E-01	-0.60	6.50E-01	-4.75	7.37E-02	-1.16	7.19E-01

med. orbital gyr.	-0.25	3.81E-01	-2.86	5.74E-02	0.04	8.09E-01	-1.92	5.84E-01
lat. orbital gyr.	-2.74	2.49E-01	-2.96	3.78E-01	-0.66	7.71E-01	-1.37	5.25E-01
post. orbital gyr.	-3.00	9.73E-02	-2.97	1.67E-01	0.07	9.17E-01	1.93	4.37E-01
subgenual frontal cort.	-6.01	1.92E-01	-10.33	2.12E-02	-0.46	9.20E-01	2.91	5.43E-01
pre-subgenual frontal cort.	-1.08	8.28E-01	-5.62	3.27E-01	-5.06	3.52E-01	0.35	7.91E-01
Occipital lobe								
lingual gyr.	-2.80	7.63E-01	-2.39	9.58E-01	-3.53	4.32E-01	-0.55	7.67E-01
cuneus	-8.22	2.29E-01	-5.83	5.68E-01	-2.83	3.75E-01	-0.47	9.50E-01
occipital lobe	-2.92	4.88E-01	-0.71	9.68E-01	-3.91	6.54E-02	-0.78	5.85E-01
Parietal lobe								
straight gyr.	-1.70	3.20E-01	-3.59	6.84E-02	0.41	7.35E-01	1.65	5.14E-01
postcentral gyr.	-6.91	6.09E-02	-4.84	3.45E-01	-9.81	5.67E-04	-5.93	3.65E-02
sup. parietal gyr.	-3.73	3.34E-01	-1.99	8.49E-01	-3.27	1.99E-01	-1.71	4.77E-01
supramarginal gyr.	-5.43	1.59E-01	-2.52	6.52E-01	0.43	9.96E-01	0.19	9.29E-01
angular gyr.	-5.38	2.45E-01	-4.30	3.81E-01	-4.90	1.08E-01	-5.05	1.12E-01
Central structures								
caudate nucleus	-3.42	2.04E-01	-2.01	4.87E-01	-3.03	3.10E-01	-0.65	7.26E-01
nucleus accumbens	-0.26	6.88E-01	-0.65	8.38E-01	-12.47	2.78E-01	-0.58	5.09E-01
putamen	-5.68	4.14E-02	-3.26	3.36E-01	-5.11	6.59E-02	-2.32	4.83E-01
thalamus	-6.52	4.94E-03	-2.27	2.28E-01	**-9.23**	**1.41E-05**	-1.63	5.31E-01
pallidum	-9.44	1.32E-01	-8.25	7.33E-02	-6.80	2.58E-01	-4.19	7.36E-01
corpus callosum (np)	-0.82	5.31E-01			-2.72	5.75E-01		
substantia nigra	-5.69	4.62E-02	-1.98	3.80E-01	-21.24	2.37E-01	-6.98	6.22E-01
Posterior fossa & ventricles								
cerebellum	-1.72	6.62E-01	-2.55	3.75E-01	-0.57	9.42E-01	-1.23	9.82E-01
brainstem (np)	-0.54	5.89E-01			2.65	1.76E-01		
lat. ventricle, main	-7.15	5.12E-01	-4.80	7.84E-01	15.26	2.48E-01	8.58	6.32E-01
lat. ventricle, temp. horn	-5.08	5.27E-01	-0.43	8.08E-01	-3.19	5.99E-01	-2.98	7.42E-01
third ventricle (np)	-13.43	1.69E-01			17.31	1.69E-01		

Supplementary Table E5. Group differences for the DKT40 atlas database (right HS).

Group differences between healthy control (HC) subjects and right hippocampal sclerosis (RHS) patients for the DKT40 atlas set with the MAPER and FreeSurfer segmentation methods. Negative percentage difference values indicate smaller volumes in RHS than HC. Significant differences in structure volumes between HC and RHS groups (from the ANCOVA, after adjustment for 62 multiple comparisons) shown in bold. Abbreviations as in Supp. Table B1.

	MAPER				FreeSurfer			
**Region**	**Right Hemisphere**		**Left Hemisphere**		**Right Hemisphere**		**Left Hemisphere**	
	**% diff**	**p-value**	**% diff**	**p-value**	**% diff**	**p-value**	**% diff**	**p-value**
Temporal lobe								
entorhinal cort.	4.18	6.23E-01	8.85	4.39E-02	-2.07	5.50E-01	-6.10	2.87E-02
parahippocampal gyr.	-9.99	1.62E-02	-0.79	9.11E-01	-0.75	7.02E-01	-7.01	1.22E-01
sup. temp. gyr.	-3.08	1.57E-01	-0.04	7.50E-01	0.55	9.29E-01	-5.30	4.37E-01
middle temp. gyr.	-6.01	1.88E-02	-1.34	6.96E-01	-1.14	6.92E-01	-4.60	1.14E-01
inf. temp. gyr.	-2.42	2.20E-01	3.93	1.56E-01	1.52	5.85E-01	-5.80	3.21E-02
fusiform gyr.	-2.89	2.71E-01	-0.03	7.74E-01	-3.07	5.58E-01	-6.27	2.96E-02
transverse temp. gyr.	5.26	3.40E-01	5.60	5.36E-01	-1.76	9.65E-01	1.67	4.86E-01
Insula and cingulate gyri								
insula cort.	-0.48	7.38E-01	1.25	1.10E-01	-1.50	9.89E-01	-2.08	4.99E-01
cingulate gyr., rostral ant.	-4.47	3.24E-01	1.46	4.76E-01	-1.80	8.75E-01	-1.93	3.40E-01
cingulate gyr., caudal ant.	3.14	2.53E-01	-0.86	7.09E-01	3.19	5.07E-01	-11.13	6.09E-04
cingulate gyr., post.	1.30	8.49E-01	1.99	9.75E-01	-0.01	8.29E-01	-4.21	2.89E-01
cingulate gyr., isthmus	0.38	6.65E-01	1.94	7.46E-01	0.96	5.86E-01	-5.24	4.73E-02
Frontal lobe								
middle frontal gyr., rostral	-1.57	5.23E-01	2.01	3.50E-01	-0.65	8.91E-01	1.64	3.43E-01
middle frontal gyr., caudal	6.46	1.76E-01	5.55	2.07E-01	-1.71	5.10E-01	-6.83	1.31E-02
sup. frontal gyr.	-0.93	6.06E-01	2.15	3.38E-01	-0.44	9.14E-01	-2.65	3.85E-01
pars opercularis	-1.51	9.25E-01	-2.50	6.26E-01	-0.84	7.15E-01	-3.48	1.78E-01
pars triangularis	0.84	5.35E-01	5.93	5.30E-02	-1.06	7.47E-01	2.71	2.12E-01
pars orbitalis	0.55	6.77E-01	-2.03	6.87E-01	-2.03	7.47E-01	-4.37	1.87E-01
lat. orbitofrontal	1.57	1.64E-01	1.49	1.65E-01	-1.37	6.20E-01	-6.08	5.59E-02
med. orbitofrontal	1.17	2.50E-01	2.52	1.35E-01	-1.89	6.25E-01	-4.54	7.20E-02
precentral gyr.	-1.73	4.14E-01	-2.15	2.91E-01	-0.63	8.36E-01	-2.28	3.23E-01
paracentral gyr.	-3.39	1.62E-01	-2.25	3.48E-01	-1.27	7.08E-01	-4.04	1.67E-01
Occipital lobe								

lingual gyr.	-0.43	9.00E-01	-0.22	8.09E-01	-1.23	6.22E-01	-4.78	6.36E-02
cuneus	-7.37	1.02E-01	-5.01	3.72E-01	-2.12	5.35E-01	-7.17	2.11E-02
lat. occipital lobe	0.81	7.89E-01	2.42	5.88E-01	-1.45	6.08E-01	-4.79	4.73E-02
pericalcarine cort.	-7.74	2.55E-01	0.65	7.62E-01	-1.07	7.53E-01	-4.11	2.63E-01
Parietal lobe								
postcentral gyr.	-4.55	1.01E-01	-0.60	9.63E-01	**1.79**	**7.86E-01**	-3.02	2.63E-01
sup. parietal gyr.	0.89	5.82E-01	0.24	9.08E-01	0.27	9.27E-01	-3.84	4.16E-01
supramarginal gyr.	-3.21	2.05E-01	-0.38	9.52E-01	-1.54	9.29E-01	-2.05	4.53E-01
inf. parietal gyr.	-3.24	2.20E-01	-1.84	5.38E-01	1.30	5.59E-01	-8.71	3.09E-02
precuneus	-4.32	7.29E-02	-3.72	2.15E-01	-0.21	8.50E-01	-2.00	3.28E-01

Supplementary Table E6. Group differences for the MGC2012 atlas database (right HS).

Group differences between healthy control (HC) subjects and right hippocampal sclerosis (RHS) patients for the MGC2012 atlas set with the MAPER and FreeSurfer segmentation methods. Negative percentage difference values indicate smaller volumes in RHS than HC. Significant differences in structure volumes between HC and RHS groups (from the ANCOVA, after adjustment for 132 multiple comparisons) shown in bold. Abbreviations as in Supp. Table B1.

	**MAPER**				**FreeSurfer**			
**Region**	**Right Hemisphere**		**Left Hemisphere**		**Right Hemisphere**		**Left Hemisphere**	
	**% diff**	**p-value**	**% diff**	**p-value**	**% diff**	**p-value**	**% diff**	**p-value**
Temporal lobe								
hippocampus	**-27.25**	**3.58E-15**	-3.19	7.31E-02	**-38.33**	**4.89E-13**	0.74	5.60E-01
amygdala	-0.06	6.72E-01	-9.39	2.97E-03	-8.66	1.21E-01	6.00	7.48E-02
entorhinal area	-4.50	2.86E-01	-10.02	9.97E-03	-13.57	1.09E-02	-7.24	8.72E-02
parahippocampal gyr.	-2.78	4.44E-01	-6.15	6.02E-03	-9.61	8.91E-03	-2.37	4.15E-01
sup. temp. gyr.	-3.33	3.88E-01	-3.04	5.26E-01	-5.37	8.13E-02	-2.47	5.10E-01
middle temp. gyr.	-7.06	1.88E-02	-2.22	6.78E-01	-4.97	4.02E-02	-2.78	2.21E-01
inf. temp. gyr.	-4.68	5.25E-02	-1.66	4.74E-01	-5.43	4.42E-02	-1.14	6.02E-01
fusiform gyr.	-2.86	4.70E-01	-0.75	4.48E-01	-5.06	1.06E-01	-0.34	9.33E-01
transverse temp. gyr.	-3.04	1.85E-01	-6.57	1.08E-01	-1.48	9.34E-01	-1.73	9.09E-01
temporal pole	-4.73	5.85E-02	-3.38	2.11E-01	-10.49	2.01E-03	-0.49	7.29E-01
planum temporale	-3.76	1.73E-01	-0.16	7.43E-01	-1.41	8.62E-01	-3.07	5.76E-01
planum polare	-4.62	4.47E-01	-3.05	7.38E-01	-4.73	1.99E-01	1.19	6.16E-01
Insula and cingulate gyri								
ant. insula	-2.16	9.78E-01	-2.58	4.42E-02	-4.14	1.35E-01	-0.61	6.72E-01
post. insula	-0.72	7.60E-01	-2.69	6.72E-01	3.38	2.38E-01	-2.48	5.78E-01
cingulate gyrus, ant.	-1.54	8.71E-01	-1.23	9.01E-01	-3.00	6.47E-01	-2.22	6.02E-01
cingulate gyrus, middle	-3.12	4.63E-01	-0.81	7.49E-01	-2.84	3.95E-01	-1.26	8.27E-01
cingulate gyrus, post.	-2.16	3.38E-01	-0.11	8.69E-01	-0.82	7.43E-01	0.14	9.46E-01
Frontal lobe								
middle frontal gyr.	-1.57	3.20E-01	-2.34	1.02E-01	-2.80	3.42E-01	-0.94	7.74E-01
precentral gyr.	-0.39	8.50E-01	-2.69	3.62E-01	-6.74	3.89E-02	-7.07	2.24E-02
precentral gyr., med.	-4.24	1.92E-01	-2.85	5.64E-01	-3.97	2.34E-01	-2.26	5.32E-01
sup. frontal gyr.	-1.87	5.40E-01	-5.66	4.34E-02	-4.92	1.20E-01	-0.27	9.98E-01
sup. frontal gyr., med.	-5.18	1.23E-01	-1.65	8.97E-01	-3.81	1.84E-01	-2.17	4.83E-01
med. frontal cort.	-0.84	4.63E-01	-2.77	2.82E-01	1.37	5.74E-01	6.50	3.33E-02

inf. frontal gyr., opercular	-0.83	7.72E-01	-3.01	7.59E-01	-2.39	4.66E-01	-5.50	2.36E-01
inf. frontal gyr., orbital	-6.24	3.27E-01	-1.24	8.88E-01	-3.79	3.93E-01	-3.97	1.62E-01
inf. frontal gyr., triangular	-2.93	1.66E-01	-5.36	5.59E-02	-4.12	3.77E-01	0.58	7.34E-01
frontal operculum	-5.46	5.22E-01	-2.72	1.87E-01	-0.13	7.87E-01	1.47	5.88E-01
frontal pole	-2.24	3.04E-01	-4.09	1.05E-01	-8.81	5.13E-02	0.38	8.96E-01
central operculum	-0.36	4.83E-01	-1.26	6.25E-01	-3.31	2.24E-01	-3.64	2.56E-01
ant. orbital gyr.	-7.63	1.05E-02	-2.60	3.58E-01	-0.34	9.11E-01	-1.55	7.44E-01
post. orbital gyr.	-0.07	8.33E-01	-1.43	8.60E-01	1.87	5.07E-01	1.58	5.08E-01
med. orbital gyr.	-0.52	6.15E-01	-1.08	2.50E-01	0.67	6.01E-01	-0.22	6.75E-01
lat. orbital gyr.	-2.29	3.76E-01	-2.46	4.15E-01	-2.09	4.97E-01	0.74	9.90E-01
supplementary motor cort.	-3.22	3.87E-01	-0.14	9.45E-01	-3.65	2.80E-01	-1.35	6.60E-01
Occipital lobe								
lingual gyr.	-0.20	5.89E-01	-0.15	4.65E-01	-4.52	1.89E-01	-1.51	5.87E-01
cuneus	-3.62	7.17E-01	-3.16	8.20E-01	-0.77	8.70E-01	1.10	6.98E-01
sup. occipital gyr.	-0.04	6.07E-01	-0.88	4.95E-01	2.34	6.77E-01	-1.11	6.45E-01
middle occipital gyr.	-3.05	5.14E-01	-2.13	4.72E-01	-9.46	1.19E-02	-7.64	2.68E-02
inf. occipital gyr.	-1.97	7.36E-01	-0.33	9.89E-01	-8.62	1.51E-02	2.02	7.92E-01
occipital pole	-0.79	8.80E-01	-1.55	6.04E-01	3.58	4.16E-01	9.63	2.45E-02
occipital fusiform gyr.	-1.82	9.28E-01	-2.49	6.93E-01	2.13	6.88E-01	0.05	9.01E-01
calcarine cort.	-5.39	5.84E-01	-1.24	8.68E-01	-3.98	5.64E-01	-0.94	8.43E-01
Parietal lobe								
gyrus rectus	-2.29	7.82E-01	-0.98	4.61E-01	0.13	7.80E-01	-1.02	7.02E-01
postcentral gyr.	-4.16	1.91E-01	-0.30	7.28E-01	-12.66	4.39E-04	-6.17	6.43E-02
postcentral gyr., med.	-8.98	2.83E-01	-1.81	4.59E-01	-10.69	4.09E-02	-8.38	1.12E-01
sup. parietal lobule	-1.24	3.07E-01	-4.14	4.07E-02	-1.10	7.51E-01	-1.17	6.41E-01
supramarginal gyr.	-4.79	2.01E-01	-1.18	8.31E-01	-3.76	1.88E-01	0.54	8.99E-01
precuneus	-3.47	4.04E-01	-3.30	4.97E-01	-4.52	7.94E-02	-2.54	3.31E-01
parietal operculum	-5.46	4.23E-02	-1.72	4.93E-01	11.87	2.29E-03	-1.16	9.53E-01
angular gyrus	-5.53	8.19E-02	-3.04	5.12E-01	-3.80	1.83E-01	-4.88	1.13E-01
Central structures								
caudate	-5.19	1.85E-01	-3.22	4.63E-01	-1.74	5.20E-01	-1.40	5.57E-01
accumbens area	-2.93	5.37E-01	-1.25	5.82E-01	-2.33	9.85E-01	-1.06	6.25E-01
putamen	-7.86	2.09E-02	-5.15	2.85E-01	-5.44	4.38E-02	-2.18	5.53E-01
thalamus (proper)	**-9.21**	**2.34E-04**	-3.51	1.34E-01	**-8.18**	**2.21E-05**	-1.25	6.83E-01
pallidum	-3.17	4.07E-01	-4.32	1.94E-01	-6.13	1.92E-01	-5.14	3.61E-01

ventral diencephalon	-2.83	1.40E-01	-1.03	9.05E-01	-2.21	2.60E-01	0.00	9.70E-01
optic chiasm (np)	-0.21	8.75E-01			-8.90	1.62E-01		
subcallosal area	-3.55	1.73E-01	-5.02	7.02E-02	-0.32	8.43E-01	0.89	8.59E-01
Posterior fossa, ventricles & white matter								
cerebellum exterior	-0.87	4.54E-01	-0.12	7.17E-01	1.09	8.03E-01	6.87	4.45E-02
cerebellar vermal lobules I-V (np)	-0.33	6.06E-01			6.48	2.34E-01		
cerebellar vermal lobules VI-VII (np)	-0.45	3.64E-01			-9.64	1.80E-01		
cerebellar vermal lobules VIII-X (np)	-1.69	7.53E-01			4.05	2.68E-01		
brainstem (np)	-0.86	5.22E-01			3.67	8.22E-02		
lateral ventricle	-12.46	2.95E-01	-6.53	6.93E-01	15.53	2.35E-01	8.69	6.08E-01
inf. lateral ventricle	-13.39	2.26E-01	-2.51	7.84E-01	19.75	4.21E-02	-3.32	3.57E-01
third ventricle (np)	-15.88	1.39E-01			14.48	2.44E-01		
fourth ventricle (np)	-0.04	7.27E-01			-1.26	8.29E-01		
cerebrospinal fluid (np)	-4.84	7.57E-01			6.75	4.50E-01		
cerebral white matter	-3.84	6.44E-03	-1.42	2.81E-01	-0.28	7.88E-01	2.49	1.94E-01
cerebellar white matter	-7.76	8.85E-02	-7.09	1.19E-01	-2.48	9.96E-01	-9.29	1.62E-01

Supplementary Figure S1. Boxplots of Jaccard overlaps for the HM atlas database.

Boxplots of JC (across 30 subjects) for each region are shown for MAPER (top) and FreeSurfer (bottom). Regions are colour coded by lobe: red – temporal lobe, orange – insula & cingulate, yellow – frontal lobe, green – occipital lobe, blue – parietal lobe, purple – central structures, pink – posterior fossa & ventricles. The top and bottom edges of the boxes are the 25th and 75th percentiles, the target inside each box is the median and outliers are indicated by unfilled circles on either end of the box whiskers. Abbreviations: L = left, R = right, gyr. = gyrus, cort. = cortex, ant. = anterior, post. = posterior, sup. = superior, inf. = inferior, med. = medial, lat. = lateral, temp. = temporal.

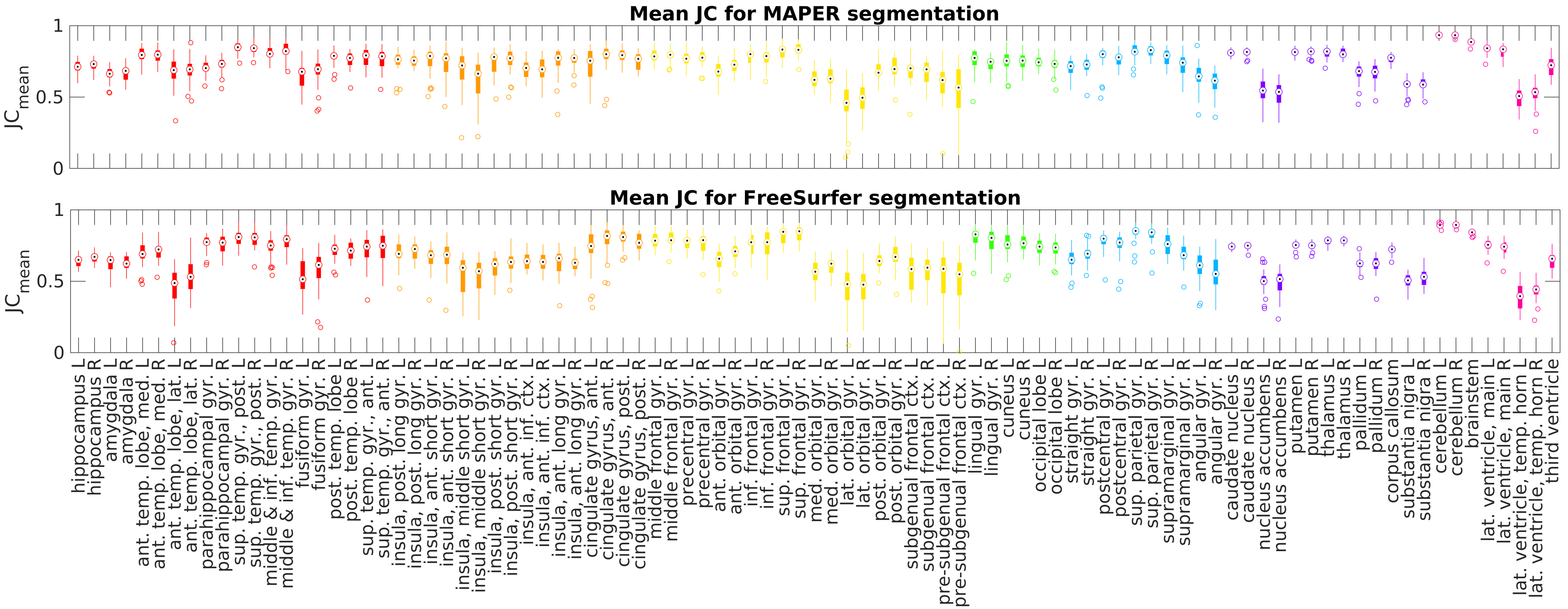

Supplementary Figure S2. Boxplots of Jaccard overlaps for the DKT40 atlas database.

Boxplots of JC (across 30 subjects) for each region are shown for MAPER (top) and FreeSurfer (bottom). Plot colours and abbreviations as in Supplementary Figure 1.

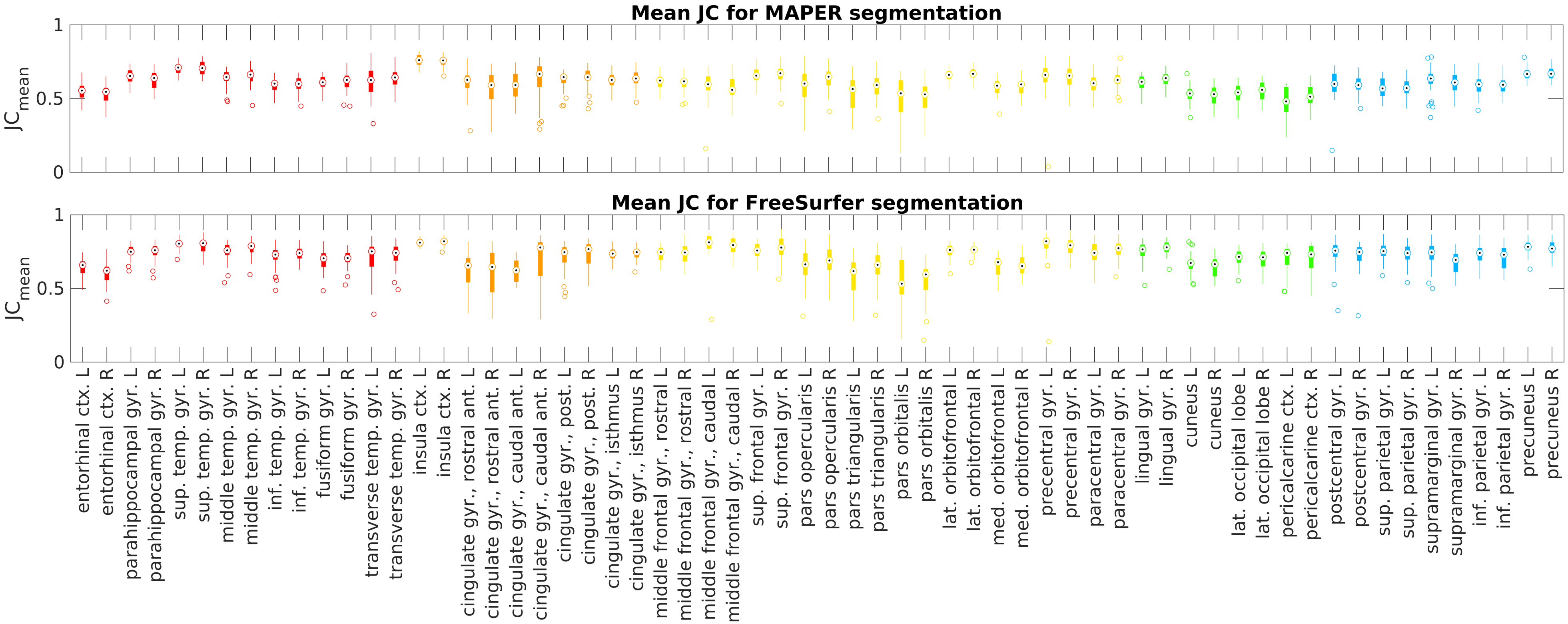

Supplementary Figure S3. Boxplots of Jaccard overlap for the MGC2012 atlas database.

Boxplots of JC (across 30 subjects) for each region are shown for MAPER (top) and FreeSurfer (bottom). Plot colours and abbreviations as in Supplementary Figure 1.

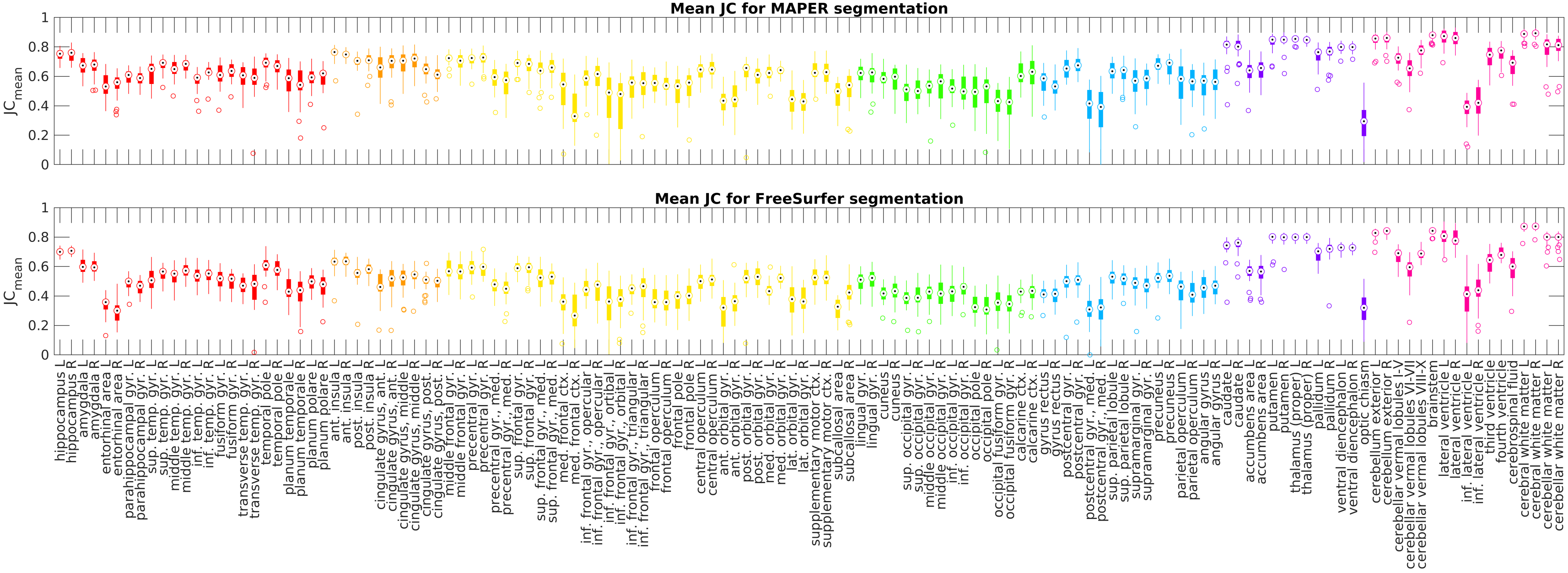

Supplement: Supplementary file 1 — Supplementary Material. [file 41598_2020_57951_MOESM1_ESM.pdf]
